# Chitosan-Based Antibacterial Bioceramic Materials for Dental Pulp Capping

**DOI:** 10.1021/acsbiomaterials.5c00466

**Published:** 2025-09-01

**Authors:** Zhi-Yi Huang, Glemarie C. Hermosa, Jyun-Sain Wu, Tung-Lin Wu, Chih-Ching Chien, Chien-Shiun Liao, Yu-Tzu Huang, Hui-Min David Wang, An-Cheng Aidan Sun

**Affiliations:** † Department of Chemical Engineering and Materials Science, 34895 Yuan Ze University, Taoyuan 320315, Taiwan; ‡ Ming-Kuan Biomedical Technology Corporation, Hsinchu 300024, Taiwan; § School of Medicine, College of Medicine, 34903 Fu Jen Catholic University, New Taipei City 242062, Taiwan; ∥ Institute of Biomedical and Pharmaceutical Sciences, School of Medicine, Fu Jen Catholic University, New Taipei City 242062, Taiwan; ⊥ Graduate Institute of Biomedical Engineering, National Chung Hsing University, Taichung 402271, Taiwan; # Graduate School of Biotechnology and Bioengineering, 197918 Yuan Ze University, Taoyuan 320315, Taiwan

**Keywords:** Tricalcium silicate, pulp-capping material, chitosan, antibacterial, biocompatibility, biofilm

## Abstract

Conventional clinical approaches for regenerative endodontic procedures, root canal therapy, and vital pulp therapy often lack sufficient antimicrobial efficacy, thereby increasing the risk of post-treatment apical periodontitis. To overcome this limitation, a series of antimicrobial powders (referred to as the AC_*S*_ series) was synthesized through a chemical reaction between tricalcium silicate (C_3_S) powder and chitosan solution pretreated with acetic acid. Following this, the AC_*S*_ powders were subsequently physically blended with additional C_3_S to enhance the mechanical properties, thereby developing a chitosan-based bioceramic composite, named the AC_*S*_-C series. The antibacterial properties of the AC_*S*_-C materials were systematically evaluated using minimum inhibitory concentration (MIC) assays, inhibition zone tests, and antibacterial assessments against *Escherichia coli* and *Streptococcus mutans* as well as biofilm testing with *Porphyromonas gingivalis*. In addition, biocompatibility was assessed through cytotoxicity tests using L929 fibroblast cells. The results revealed that the AC_20_-C formulation exhibited good antibacterial efficacy (exceeding 90%), maintained over 80% cell viability, exhibited a clear inhibition zone, and effectively inhibited biofilm formation. Regarding physical properties, the AC_*S*_-C materials were able to set within 30 min and possessed sufficient compressive strength. Further structural analysis using energy-dispersive X-ray spectroscopy, X-ray diffraction, and Fourier transform infrared spectroscopy verified the successful synthesis and structural integrity of the material. The AC_*S*_-C samples exhibited key functional groups, including amino, amide, Si–O, CaO, and PO_4_^3–^. In this study, the AC_*S*_-C series represent promising antimicrobial bioceramic pulp-capping materials that combine effective antibacterial activity with favorable biocompatibility.

## Graphical abstract

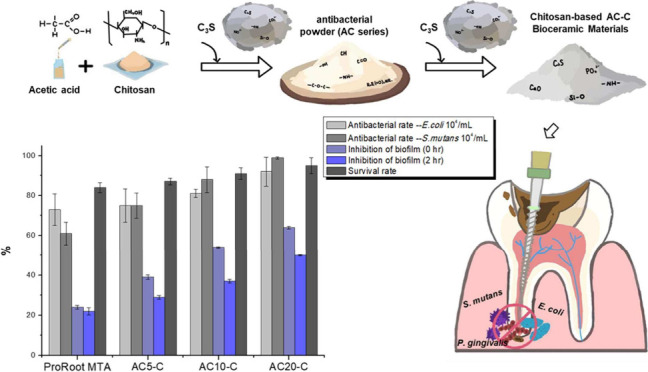

## Introduction

The dental pulp, derived from the neural crest mesenchymal tissue, is located at the core of the tooth, where it nourishes and protects the surrounding dentin. However, when the pulp is compromised due to caries or trauma, its regenerative capacity is diminished, leading to pulpitis. Clinical interventions, such as root canal therapy, apexification, and vital pulp therapy are designed to restore dentin and preserve tooth function. The development of materials for root canal fillings began in the early 1970s. However, these early materials were associated with a high recurrence rate of infection, reported to range from 50% to 90%. Subsequent advances in biomaterials research have led to the development of new capping materials, such as ProRoot mineral trioxide aggregate (ProRoot MTA, Dentsply Tulsa Dental, Tulsa, OK, USA) and Biodentine (Septodont, St. Maur-des-Fossés, France). Despite these innovations, clinical studies have indicated that these materials may still demonstrate limited antibacterial performance. Incomplete sealing of deep root canals may allow residual bacteria to persist, leading to reinfection and eventual treatment failure.^1–5^ These issues underscore the need for bioactive materials with improved antibacterial functionality.

The antimicrobial activity of pulp-capping materials plays a crucial role in determining treatment outcomes. Ideal materials should possess both suitable flowability and the capacity to eliminate residual microorganisms near the exposed pulp while minimizing damage to surrounding tissue.^6^ Common endodontic materials, including calcium hydroxide (Ca­(OH)_2_) and tricalcium silicate (Ca_3_SiO_5_, C_3_S)-based aggregates (e.g., MTA and C_3_S), are bioactive ceramics that can induce the formation of calcium bridges at pulp exposure sites. ProRoot MTA is well-recognized for its biocompatibility and its ability to promote tissue mineralization. However, previous studies^7–10^ employing mechanical pulp exposure models have not adequately replicated clinical inflammatory conditions, limiting the applicability of the findings to scenarios involving chronic inflammation or reinfection. Biodentine shares similar characteristics with ProRoot MTA, but offers advantages such as improved handling properties, shorter setting time, and higher mechanical strength. Furthermore, Biodentine has demonstrated the capacity to inhibit several bacterial strains, including *Streptococcus mutans*, *Escherichia coli*, *Enterococcus faecalis*, and *Candida albicans*.^11–13^ Nonetheless, its antimicrobial performance remains inconclusive due to inconsistent outcomes in biofilm-related studies. Previous reports have identified differences in minimum inhibitory concentration (MIC) values and suspension turbidity between ProRoot MTA and Biodentine, likely influenced by the alkaline environment generated during calcium silicate hydrolysis. However, this chemical environment does not fully replicate the biofilm conditions typically encountered in clinical settings.^14^

The aforementioned findings highlight continued debates regarding the antibacterial reliability of commercially available endodontic materials. In response to these challenges, the present study developed a novel antimicrobial pulp-capping material (AC_*S*_-C compound) through a physicochemical blending of C_3_S and chitosan-based biopolymers. Chitosan, a polysaccharide obtained via the deacetylation of chitin, exhibits broad-spectrum antibacterial activity and is soluble only under mildly acidic conditions. It has demonstrated inhibitory effects against both Gram-positive and Gram-negative bacteria.^7,15–19^ However, most existing formulations tend to emphasize either antimicrobial action or osteogenic potential, and few have successfully integrated both properties into a single composite system. In contrast, the AC_*S*_-C compound developed in this study was designed to address endodontic bacterial contamination while simultaneously supporting cell proliferation within 24 h. Calcium silicate-based cements interact with phosphate ions to form apatite layers, thereby improving the sealing performance of the material. This interaction also enhances cytokine release by osteoblasts, which facilitates the formation of thicker dentin bridges and stimulates stem cell proliferation.^10,11,20–22^ Building on a previous study^23^ that synthesized Ca_3_SiO_5_ powder using a sol–gel method, the present study further incorporated antimicrobial powder into C_3_S-rich composites to increase calcium ion release.

The present study focused on three clinically relevant oral pathogens: *E. coli*; *S. mutans*, known to cause dental caries and endocarditis; and *P. gingivalis*, a key pathogen in chronic periodontitis. The antibacterial activity of the newly synthesized capping materials was compared with that of ProRoot MTA and Biodentine.^24^ Additionally, the biocompatibility of the materials was evaluated through cytotoxicity assays, and their physicochemical characteristics were analyzed using energy-dispersive X-ray spectroscopy (EDX), X-ray diffraction (XRD), Fourier transform infrared spectroscopy (FTIR), and mechanical testing with a universal testing machine.

## Materials and Methods

### Preparation of Antibacterial Dental-Pulp-Capping Materials

#### Chitosan Solution Preparation

To prepare chitosan formulations, we tested various concentrations to determine the optimal conditions. Chitosan (Emperor Chemical Co., Ltd., Taipei City, Taiwan) was dissolved in an acetic acid solution, and the synthesis process was conducted at 90 °C under continuous stirring for 4 h. The acetic acid concentration was gradually increased, with the chitosan concentration was kept at 2%. After testing different formulations, we determined that a mixture of 2% chitosan acetic acid solution (2% chitosan in 30% acetic acid solution, named Chitosan Solution) provided the most effective conditions for synthesizing antimicrobial powders.

#### Preparation of AC_*S*_-Series (AC_*S*_) Chitosan-Based Antibacterial Materials

Chitosan, chosen for its antibacterial properties, was incorporated as the key antimicrobial component. After being reacted with acetic acid, chitosan solution was combined with C_3_S powder (average particle size ∼ 500 nm, confirmed to have uniform distribution via dynamic light scattering (DLS); Ming-Kuan Biomedical Technology Co., Ltd., Hsinchu County, Taiwan) to create an antimicrobial powder referred to as the AC_*S*_-Series (AC_*S*_). As shown in Table 1, the chitosan-based AC_*S*_ biomaterials were prepared by mixing with 1.8 g of C_3_S powder and various amount of chitosan solution (1.52–7.6 mL) until a cohesive mixture was achieved. The mixture was then dried at 80 °C to remove moisture and facilitate the formation of chemical bonds. After drying, the AC_*S*_ powders were ground and sieved to ensure uniform particle size. The antibacterial activity of AC_*S*_ powders was evaluated, and formulations that demonstrated over 99% antibacterial performance (AC_4_, AC_5_, AC_10_, and AC_20_) were selected for further investigation.

**Table 1:**
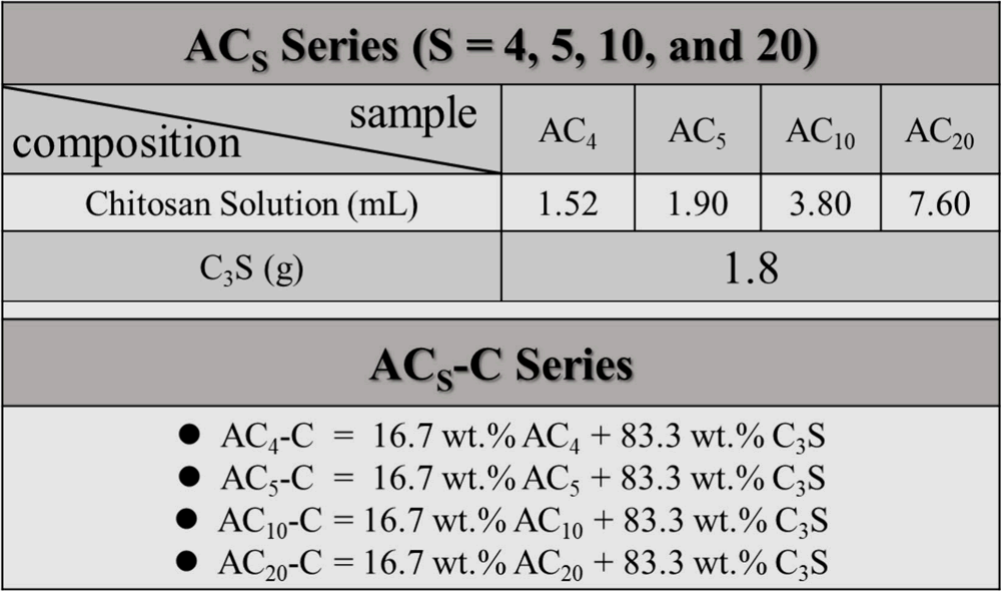
Experimental Sample Parameters

#### Synthesis of Chitosan-Based Composite Biomaterials (AC_*S*_-C Series)

To enhance the calcium ion content in the pulp-capping material, the previously developed antimicrobial powders (AC_4_, AC_5_, AC_10_, and AC_20_) were physically mixed with additional C_3_S powder at a fixed ratio of 83.3%, as shown in Table 1. This composition was established based on our earlier research involving the incorporation of Ta_2_O_5_ into the C_3_S system, which demonstrated that maintaining 83.3% C_3_S achieves an optimal balance between rapid setting and sufficient compressive strength due to its high calcium content. For example, in a total powder mass of 0.6 g, a mixture of 0.1 g of AC_*S*_ and 0.5 g of C_3_S corresponds to this ratio, where AC_*S*_ constitutes one-sixth of the total mass. This proportion results in 16.7% AC_*S*_ and 83.3% C_3_S by weight. Through this formulation strategy, a new series of composite materials was developed, designated as AC_4_-C, AC_5_-C, AC_10_-C, and AC_20_-C. These materials were designed to retain effective antimicrobial activity while simultaneously improving the setting behavior and mechanical performance through increased calcium availability.

Leite et al.^25^ also utilized chitosan in their study, focusing on the development of calcium silicate-coated porous chitosan scaffolds for pulp tissue engineering. However, their approach differs significantly from ours in both material design and intended clinical application. While their study employed commercial calcium silicate powders and relied on freeze-drying to produce scaffolds suitable for indirect use, our research developed a clinically applicable, powder-based pulp-capping material by combining sol–gel-synthesized tricalcium silicate (C_3_S) nanoparticles with antimicrobial chitosan-based powders. This formulation allows for direct chairside use without the need for freeze-drying or chemical cross-linking. Furthermore, our study emphasizes not only the physical and chemical properties of the material but also its early stage antibacterial performance, biofilm inhibition capacity, and cytocompatibility-key functional requirements for effective direct pulp capping in clinical settings.

### Biological Characterization of Antibacterial Pulp-Capping Biomaterials

#### Antibacterial Assay against *E. coli* and *S. mutans*

To evaluate the antibacterial properties of the dental-pulp-capping materials, antibacterial assays were performed. Samples were first dissolved in phosphate-buffered saline (PBS) (137 mM NaCl, 2.7 mM KCl, 10 mM Na_2_HPO_4_·2H_2_O, and 2.0 mM KH_2_PO_4_) at a concentration of 200 mg/mL. After 24 h, the PBS was replaced with fresh PBS. This process was repeated, and collected PBS solutions from each exchange were used as the experimental culture medium. Bacterial cultures of *E. coli* or *S. mutans* (ATCC 25175) were reactivated in their respective culture media (LB for *E. coli* and Brain Heart Infusion (BHI) for *S. mutans*; Bionovas Biotechnology Co., Ltd., Toronto, Ontario) and incubated at 37 °C with shaking at 120 rpm for 2 h. For the antibacterial assay, 400 μL of fresh culture medium, 400 μL of the experimental culture medium, and 200 μL of bacterial suspension (at 10^4^ colony-forming units [CFU]/mL or 10^6^ CFU/mL) were mixed in sterile centrifuge tubes and incubated at 37 °C with shaking at 120 rpm. After 24 h, 100 μL of the *E. coli* suspension was spread onto LB agar plates, and *S. mutans* was spread onto BHI agar plates. The plates were incubated at 37 °C for 24 h for *E. coli* and 48 h for *S. mutans*, respectively. PBS that had not been exposed to any materials served as a positive control for CFU quantification. The antibacterial activity was expressed as the percentage of bacterial inhibition (mean ± standard deviation, *n* = 3) and statistically analyzed using one-way and two-way analysis of variance (ANOVA), followed by Scheffé’s post hoc test to compare the antibacterial effects of the tested materials.^26^

#### Inhibition Zone Assay against *E. coli* and *S. mutans*

Inhibition zone testing can be used to evaluate the antibacterial activity of dental-pulp-capping materials by measuring the area around the material where bacterial growth is inhibited. The size of the inhibition zone depends on the diffusion capacity of the antibacterial agents from the test materials, the extracellular MIC, and the binding affinity to bacterial cell receptors.^27^ In this experiment, bacterial colonies of *E. coli* and *S. mutans* were cultured on agar plates. Small wells, approximately 6 mm in diameter, were created in the agar using a pipet. The test materials, AC_5_-C, AC_10_-C, and AC_20_-C, were prepared by physically mixing with 83.3% C_3_S powder, as described in Table 1. These mixtures were then introduced into the wells. ProRoot MTA was used as a control material. The diameters of the inhibition zones around the each material were measured after 24 h for *E. coli* and after 48 h for *S. mutans* to assess the antibacterial performance of the tested materials.

#### Biofilm Inhibition and Disruption Assay against *P. gingivalis*

We examined the ability of the synthesized antibacterial materials and ProRoot MTA to inhibit the formation of biofilms and to disrupt both immature and mature biofilms of *P. gingivalis* (ATCC 33277), thereby preventing high-density bacterial adhesion to 96-well polystyrene plates.^28^ Prior to the experiment, the materials were soaked in Tris-buffered saline (TBS, Bionovas Biotechnology Co., Ltd., Toronto, Ontario) at a concentration of 200 mg/mL. After 24 h of soaking, the TBS solution was used as the experimental medium.

To assess biofilm formation inhibition, 50 μL of *P. gingivalis* culture (cultured for 48 h, OD_600_ = 0.4) was added to each well of a 96-well microtiter plate (GUNSTER, New Taipei City, Taiwan), along with 100 μL of fresh TBS medium and 50 μL of the test material or control solution, bringing the final volume to 200 μL per well. Wells containing only culture medium and sample immersion solution served as positive controls, while those without the immersion solution served as negative controls. The plates were incubated anaerobically at 37 °C for 48 h. Following incubation, the medium was carefully removed, and unattached bacteria were rinsed off with 200 μL of PBS. The remaining biofilm was fixed with 200 μL of 99% methanol for 15 min, stained with 200 μL of 1% crystal violet for 5 min, and rinsed with distilled water. Finally, 200 μL of 33% glacial acetic acid was added to dissolve the bound dye, and the extent of biofilm formation, with or without treatment, was quantified using an enzyme-linked immunosorbent assay (ELISA) reader (BioTek, Winooski, USA) at 600 nm.

The same procedure was used to evaluate the disruption of biofilms at different development stages. For immature biofilms (preformed for 0–2 h) and mature biofilms (preformed for 6–24 h), *P. gingivalis* (OD_600_ = 0.4) was first added to the wells along with 100 μL of fresh TBS medium. After 2, 6, and 24 h of incubation, 50 μL of the test materials or control solution was added to assess the capacity of the pulp-capping materials to disrupt the existing biofilms. Biofilm disruption was quantified using crystal violet staining by following the same procedure used for biofilm inhibition.

#### Cytotoxicity and Cell Viability Assay

The MTT assay is a widely used method for assessing *in vitro* cytotoxicity based on the conversion of MTT into deep purple-blue formazan crystals by viable cells, which reflects mitochondrial activity. This conversion can be quantified using spectrophotometry.^29,30^ For the cytotoxicity assessment, the test samples were dissolved in Dulbecco’s Modified Eagle’s Medium (DMEM) (Gibco, Waltham, Massachusetts, USA) at a concentration of 200 mg/mL. After 24 h, the DMEM solution was collected, and the tubes were replenished with fresh DMEM to repeat the soaking process. The retrieved DMEM was used as the experimental culture medium. L929 fibroblasts were adjusted to the desired concentration in DMEM supplemented with 10% fetal bovine serum (D10; containing 1% PSA, 1% glutamine, and 10% serum by volume). The cells were seeded in a 96-well plate at a density of 1 × 10^5^ cells per 200 μL and incubated at 37 °C for 24 h. After incubation, 100 μL of the cell supernatant was removed and replaced with 100 μL of the experimental culture medium. PBS that had not been exposed to any test samples was used as the negative control. The MTT assay was then performed after 24 h of incubation at 37 °C. To conduct the assay, the wells were washed with 100 μL of PBS. Then, MTT reagent from the MTT kit (Roche, Boston, USA) was diluted in D10 at a ratio of 1:10, and 100 μL of the resulting solution was added to each well. The plate was incubated at 37 °C for 2 h to allow for formazan crystals formation. After incubation, the D10 medium was removed, and 100 μL of DMSO was added to each well to dissolve the crystals. The absorbance was measured at 570 nm by using an ELISA reader. All results were expressed as the mean ± standard deviation with three replicates (*n* = 3).

### Physicochemical Characterization of Antibacterial Dental-Pulp-Capping Materials

#### Characterization of Synthesized Antibacterial Pulp-Capping Materials

X-ray diffraction (XRD) patterns were analyzed to confirm the crystalline phases present in the synthesized materials. In accordance with ISO 9917-1:2007 standard,^31^ the materials were prepared by mixing and casting into molds with a diameter of 10 mm and a height of 1 mm. These samples were then soaked in ultrapure water at 37 °C for 7 days, followed by drying prior to testing.^32^ The surface morphology of the C_3_S powder, antimicrobial powders (AC_*S*_), and antimicrobial pulp-capping materials (AC_*S*_-C) were examined by scanning electron microscopy (SEM) on a Hitachi S-4800 microscope operating at 15 kV. The elemental composition before and after synthesis was assessed using an energy-dispersive X-ray (EDX) analyzer (RONTEC, Massachusetts, USA). FTIR was performed to identify the functional groups present in the chitosan-based biomaterial, C_3_S, antimicrobial powders, and antimicrobial pulp-capping materials. For this analysis, powder samples were immersed in simulated body fluid (PBS) for 7 days and then analyzed using a KBr pellet-based spectrometer (Germany).^33^

#### Assessment of Setting Time and Compressive Strength

The setting behavior of the antibacterial dental-pulp-capping materials was evaluated in accordance with ISO 6876:2012 standards.^34^ The materials were mixed with ultrapure water in a ratio of 0.6 g to 2.8 mL and then placed into a 2 mm in height and 10 mm in diameter acrylic mold. The samples were incubated at 37 °C with 95% relative humidity for 18 to 30 min. The initial setting time was first checked at 4 min and subsequently measured 2 min intervals. A 1 mm diameter indenter from the Gillmore needle apparatus (GNA), loaded with 0.6 g of material, was applied perpendicularly to the surface of the sample every 2 min. The initial setting time was recorded when the indenter no longer produced an indentation.

Compressive strength was evaluated following ISO 9917-1:2007 standards^31^ using a universal testing machine (HT-2402) operating at a crosshead speed of 1 mm/min. The materials were mixed with water and cast into cylindrical molds with a diameter of 4 mm and a height of 6 mm. The specimens were incubated at 37 °C with 95% relative humidity for either 7 or 28 days. After incubation, the specimens were consolidated and flattened prior to testing. The maximum force required to fracture the sample was recorded, and the compressive strength (σ) was calculated in megapascals (MPa) by using the formula σ = 4*F*/π*D* ^2^, where *F* is the maximum load in newtons (N) and *D* is the diameter of the sample in millimeters.

## Results

### Biological Evaluation

#### Antibacterial Activity and *In Vitro* Biocompatibility of Pure C_3_S and Commercial Pulp-Capping Materials

In line with the recommendations of the American Association of Endodontists, new dental materials should be developed based on sound biological principles and established clinical practices.^12,35^ Because of uncertainties regarding the antibacterial efficacy of pulp-capping materials,^23^ the present study compared the antibacterial performance of commercial dental materials with that of synthesized C_3_S through *in vitro* tests against *E. coli* and *S. mutans*. During the experiment, the samples were exposed to *E. coli* suspensions at concentrations of 10^4^ CFU/mL and 10^6^ CFU/mL for 24 h.

As depicted in Figure 1A, the synthesized C_3_S (1D) demonstrated strong antibacterial activity at both concentrations, surpassing all other tested samples. Antibacterial rates were calculated using CFU counts, and PBS serving as a negative control (mean ± standard deviation, *n* = 3). After logarithmic transformation (log_10_), the results indicated that ProRoot MTA exhibited more than 15% greater antibacterial efficacy than Biodentine did. Statistical analysis using ANOVA revealed that C_3_S (1D) showed significant differences at both bacterial concentrations compared with the commercial products ProRoot MTA and Biodentine (*P* < 0.01). By contrast, C_3_S (5D) did not exhibit any measurable antibacterial activity. When tested against *S. mutans*, ProRoot MTA demonstrated greater antibacterial activity than Biodentine, although its efficacy did not exceed 60%. Furthermore, C_3_S (1D) demonstrated significantly higher antibacterial activity at high concentrations (10^6^ CFU/mL) than ProRoot MTA (*P* < 0.01). At low test concentrations (10^4^ CFU), ProRoot MTA and Biodentine both showed statistically significant differences (*P* < 0.001 and *P* < 0.01, respectively). However, C_3_S (5D) displayed weaker antibacterial activity than both commercial materials.

**Figure 1:**
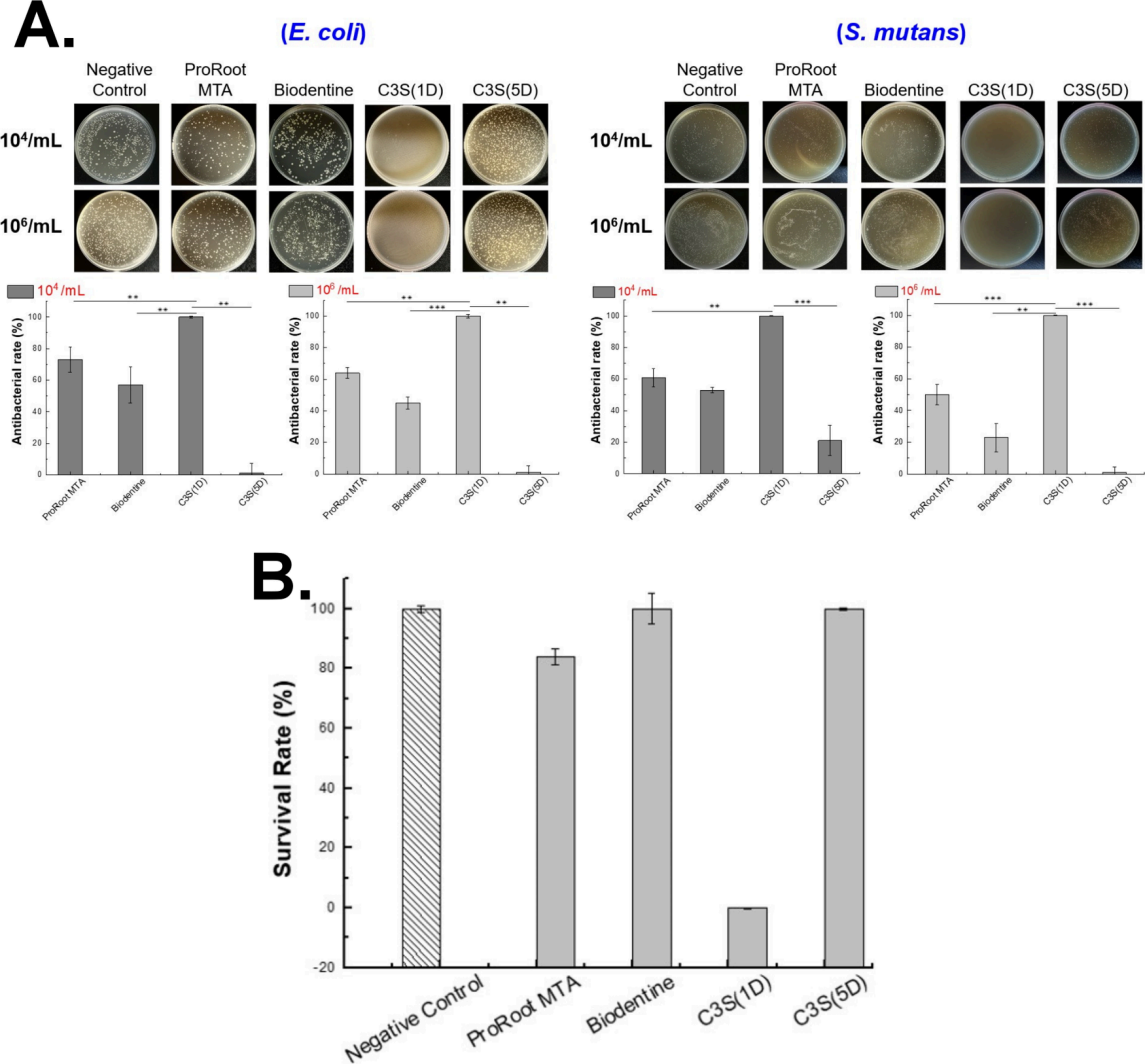
Antibacterial and biocompatibility assays of C_3_S and commercial dental materials. (A) The antibacterial efficacy of tricalcium silicate powders (C_3_S (1D) and C_3_S (5D)) was evaluated after 24 h of contact with *E. coli* and *S. mutans* at suspension concentrations of 10^4^ CFU/mL and 10^6^ CFU/mL, respectively. The performance of these powders was compared with that of commercial products (ProRoot MTA and Biodentine). (B) Biocompatibility testing was conducted using L929 fibroblasts at a concentration of 1 × 10^5^ cells/100 μL to assess cytotoxicity. The cell viability results were then compared with those obtained for the two commercial products (ProRoot MTA and Biodentine).

The reduced antibacterial effect of C_3_S (5D), when compared to C_3_S (1D) and the negative control, is likely due to a time-dependent decrease in alkalinity. Freshly synthesized C_3_S undergoes hydration, releasing calcium and hydroxide ions, thereby creating a highly alkaline environment. This alkaline condition plays a crucial role in disrupting bacterial membranes and inhibiting microbial growth.^36,37^ However, prolonged immersion and repeated replacement of medium gradually weaken this alkaline environment, leading to diminished antibacterial activity in the C_3_S (5D) group. Collectively, these results suggest that none of the tested commercial root-end filling materials achieved greater than 90% antibacterial efficacy against *E. coli* and *S. mutans*. In addition, ProRoot MTA has been reported to be ineffective in resolving infection and inflammation in cases of chronic diffuse pulpitis.^7–9^ By contrast, the newly synthesized C_3_S (1D) exhibited remarkably high antibacterial activity, underscoring its potential advantages over existing commercial materials.

To examine cell viability, mitochondrial activity was measured using the MTT assay. The results (Figure 1B) indicated that ProRoot MTA and Biodentine maintained cell viability above 80%, suggesting minimal cytotoxicity. By contrast, the newly synthesized C_3_S materials (C_3_S­(1D) and C_3_S­(5D)) exhibited significantly higher cytotoxicity, with cell viability dropping below 80% and a notable increase in cell death. Previous studies^38–40^ have reported that hydration of C_3_S leads to an elevated pH, which can damage cell membranes, interfere with cellular physiological functions, and inhibiting cell proliferation, ultimately resulting cytotoxic effects.

#### Antibacterial and Biocompatibility Evaluation of Novel Synthetic AC_*S*_ Powders

Chitosan and its derivatives exert antibacterial effects by adsorbing onto bacterial cell surfaces, disrupting the lipid membrane, and ultimately causing cell death.^41,42^ We incorporated chitosan into the synthesized C_3_S powder to enhance its antibacterial properties. The results indicated that the newly synthesized calcium silicate-chitosan composites (antimicrobial powders), consisting of chitosan blended with C_3_S in specific ratios (AC_4_, AC_5_, AC_10_, and AC_20_), demonstrated significantly stronger antibacterial activity against *E. coli* compared to ProRoot MTA (Figure 2A). These compounds completely inhibited *E. coli* growth, even at a high concentration of 10^6^ CFU/mL.

**Figure 2:**
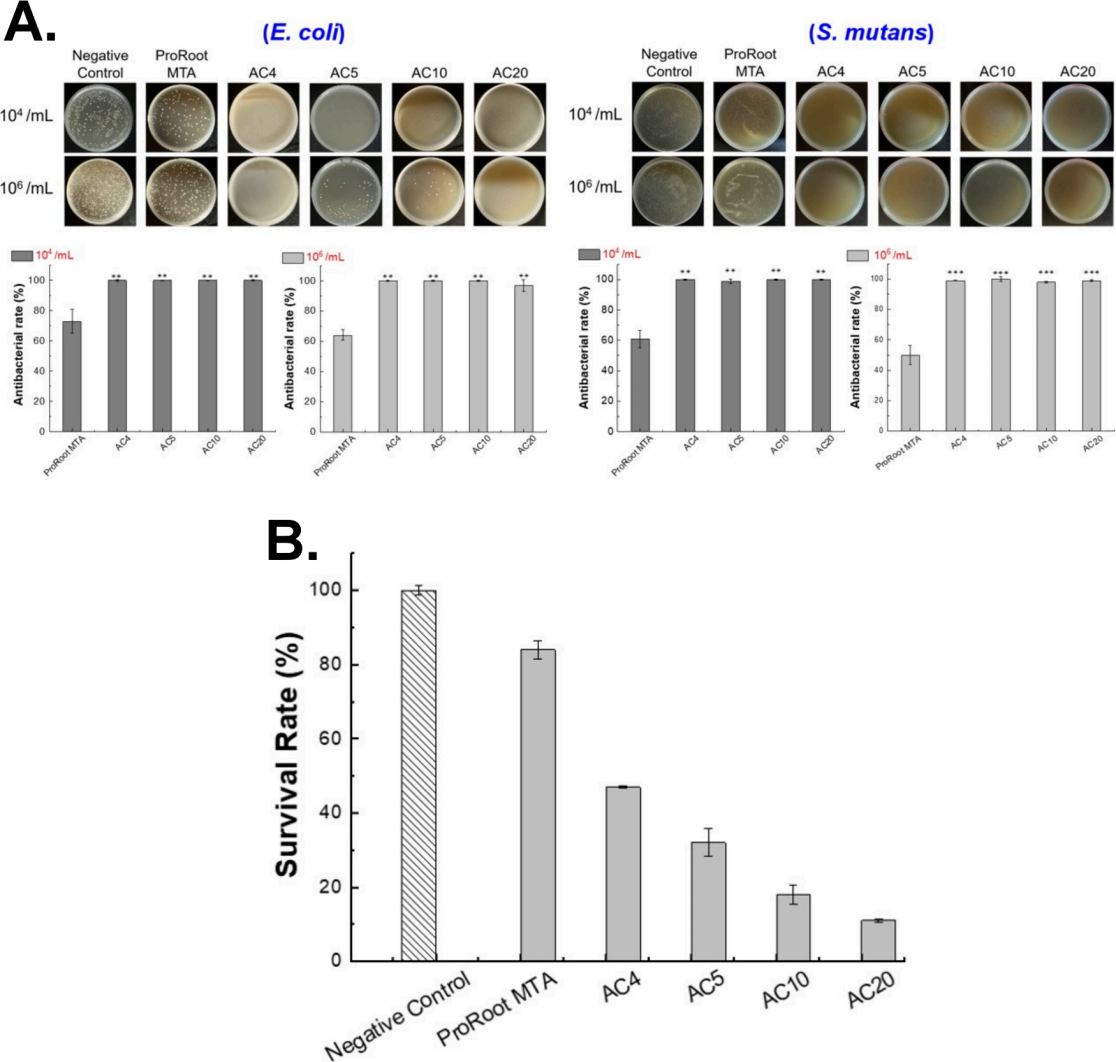
Antibacterial and biocompatibilty assays of AC_*S*_. (A) The antibacterial properties of four mixtures (AC_4_, AC_5_, AC_10_, and AC_20_) were tested after a 24 h reaction with suspension cultures of *E. coli* and *S. mutans* (10^4^ CFU/mL, 10^6^ CFU/mL). The antibacterial rate was calculated on the basis of CFU counts compared with the PBS control. Statistical significance was determined using a *t* test: *P < 0.05, **P < 0.01, ***P < 0.001, *n* = 3. (B) Biocompatibility testing was conducted to assess the cytotoxicity of the antibacterial materials (AC_20_, AC_10_, AC_5_, and AC_4_) by using L929 fibroblasts at a concentration of 1 × 10^5^ cells/100 μL.

In indirect contact tests with *S. mutans*, all synthesized AC_*S*_ compounds (AC_4_, AC_5_, AC_10_, and AC_20_) completely inhibited bacterial growth, substantially outperforming ProRoot MTA (Figure 2A). The enhanced antibacterial effect is attributed to electrostatic interactions between amino groups of chitosan and bacterial cell walls, which destabilize the membrane and lead to cell death. However, its effectiveness can vary depending on compound structure and environmental conditions.^19,41,43^

*In vitro* models can be used to evaluate the biocompatibility of biodegradable chitosan and its derivatives, particularly in identification of the potential toxic effects of residual monomers, catalysts, and other polymer-related factors.^44^ Because these models simulate biological systems, they can be used to evaluate inflammatory, immune, and mutagenic responses in practical applications. In the present study, the results of the MTT assay (Figure 2B) revealed that although the antimicrobial powders exhibited excellent antibacterial properties, varying the concentration of chitosan did not fully mitigate the cytotoxic effect of high pH on cell viability. High pH levels can compromise cell membrane integrity and metabolic activity, reducing cell survival to below 80%. However, studies^45,46^ have indicated that an alkaline environment may enhance mineralization in human dental pulp cells and promote bone regeneration in human bone marrow stromal cells by facilitating osteoblast differentiation, encouraging mineral deposition, and reducing the inhibitory effects of acidic byproducts. Therefore, future studies should focus on refining physical blending techniques to mitigate high alkalinity while preserving conditions that supports mineralization.

#### Biocompatibility and Antibacterial Performance of C_3_S-Based Composite Powders (AC_*S*_-C Series) for Pulp Capping

To ensure that incorporating C_3_S into synthesized materials does not compromise their antimicrobial effectiveness, we conducted indirect antimicrobial assays on the antimicrobial pulp-capping material. The composite calcium silicate-based materials created by blending the antimicrobial powders with C_3_S exhibited superior antibacterial performance in direct contact assays against *E. coli* at both high (10^6^ CFU/mL) and low (10^4^ CFU/mL) bacterial concentrations compared with the control (Figure 3A). Although the antimicrobial pulp-capping material, AC_5_-C, AC_10_-C, and AC_20_-C, were formulated by further physically mixing the AC_*S*_ antimicrobial powders with 83.3% C_3_S to enhance calcium ion content, they could not completely inhibit *E. coli* growth. However, compared with ProRoot MTA, these formulations demonstrated significantly improved antibacterial efficacy. At a low bacterial concentration (10^4^ CFU/mL), AC_20_-C achieved an antibacterial rate exceeding 95% (*P* < 0.01), while AC_10_-C and AC_5_-C exhibited approximately 80% efficacy. Even at a higher bacterial concentration (10^6^ CFU/mL), these composites outperformed ProRoot MTA, with AC_20_-C maintaining an antibacterial rate above 90% (*P* < 0.001) and AC_5_-C and AC_10_-C achieving antibacterial rates of 72% and 85%, respectively. These findings indicate that the newly developed AC_*S*_-C antimicrobial bioceramic-particularly AC_20_-C, significantly surpassed ProRoot MTA in antibacterial performance against *E. coli*.

**Figure 3:**
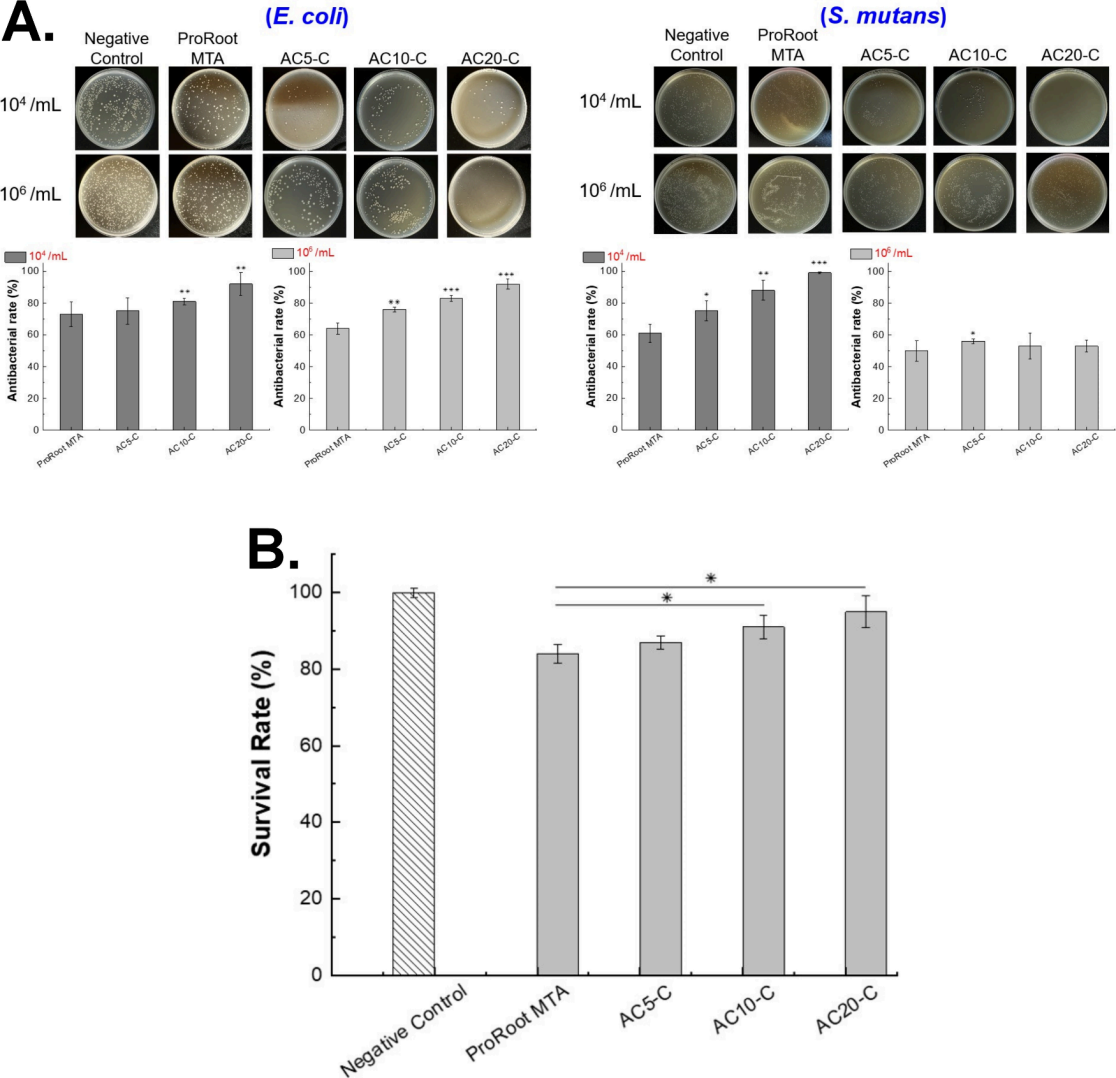
Antibacterial and cytotoxicity assays of advanced AC_*S*_-C. (A) The antibacterial properties of synthetic antibacterial materials mixed with C_3_S (AC_5_-C, AC_10_-C, and AC_20_-C) were tested using suspension cultures of *E. coli* and *S. mutans* at concentrations of 10^4^ CFU/mL and 10^6^ CFU/mL. The antibacterial rate was calculated on the basis of CFU counts compared with the PBS control. Statistical significance was determined using a *t* test: *P < 0.05, **P < 0.01, ***P < 0.001, *n* = 3. (B) Biocompatibility testing was conducted to assess the cytotoxicity of pulp antibacterial materials mixed with C_3_S (AC_20_-C, AC_10_-C, AC_5_-C, and AC_4_-C) by using L929 fibroblast cells at a concentration of 1 × 10^5^ cells/100 μL. Statistical significance was determined using a *t* test: *P < 0.05, *n* = 3.

Indirect contact testing was conducted using Gram-positive *S. mutans* (Figure 3A). The results, presented as CFU/mL after logarithmic transformation (log_10_), revealed that AC20-C exhibited strong antibacterial activity at a low bacterial concentration (10^4^ CFU/mL) (*P* < 0.001). AC_10_-C demonstrated 88% antibacterial efficacy (*P* < 0.01). Although AC_5_-C exhibited inhibition below 80%, all synthesized materials outperformed ProRoot MTA. However, under the high bacterial concentration condition (10^6^ CFU/mL), neither the AC_*S*_-C materials nor ProRoot MTA demonstrated satisfactory antibacterial efficacy.

Due to concerns that high pH may contribute to cytotoxic effects, and given the reduced calcium content in the antimicrobial powders, C_3_S was reintroduced via physical blending to achieve an improved balance. Both Ca­(OH)_2_ and C_3_S are known to stimulate dental pulp cell proliferation and promote extracellular matrix mineralization. These compounds can also induce osteoblasts to release cytokines, which interact with tissue fluid to form Ca­(OH)_2_ and facilitate the development of thicker dentin bridges.^21,47^ When C_3_S reacts with water, it releases ions that combine with phosphate ions in saliva to form hydroxyapatite (HAp), thereby enhancing sealing ability, dentin regeneration, and stem cell activation.^48–50^

The results from the MTT assay (Figure 3B) showed that combining varying concentrations of antimicrobial powders with C_3_S enhanced cell viability (above 80%) compared with ProRoot MTA, suggesting improved biocompatibility. Among the tested materials, AC_20_-C demonstrated the highest cell viability, exceeding 90% (*P* < 0.05). AC_10_-C and AC_5_-C also exhibited favorable cell biocompatibility, with cell viability ranging between 80% and 90%. When comparing data from Figures 2B and 3B, the AC_*S*_-C composite materials showed no significant cytotoxicity within 24 h, as demonstrated by the MTT assay testing on L929 fibroblasts. This favorable response indicates good initial biocompatibility. However, as the antibacterial material concentration decreased, a corresponding reduction in cell viability was observed.

#### Inhibition Zones of AC_*S*_-C Dental-Pulp-Capping Materials

Inhibition zone tests were conducted to simulate direct bacterial contact in the root canal. The AC_*S*_-C series materials exhibited larger inhibition zones against *S. mutans* than against *E. coli* at the MIC, indicating superior effectiveness against *S. mutans*. As depicted in Figure 4, AC_20_-C exhibited an inhibition zone diameter of 4.2 ± 0.25 mm for *E. coli* and 10.8 ± 0.29 mm for *S. mutans*. By contrast, ProRoot MTA exhibited significantly smaller zones, measuring 0.08 ± 0.00 mm for *E. coli* and 5.8 ± 0.29 mm for *S. mutans*. These results suggest that AC_20_-C is more than twice as effective as ProRoot MTA in inhibiting bacterial growth. In addition, other composite AC_*S*_-C series, such as AC_5_-C and AC_10_-C, also demonstrated larger inhibition zones compared to ProRoot MTA. In particular, AC_5_-C exhibited zones of 3.4 ± 0.46 mm for *E. coli* and 8.2 ± 0.76 mm for *S. mutans*, while AC_10_-C showed zones of 3.9 ± 0.07 mm for *E. coli* and 9.6 ± 1.06 mm for *S. mutans*. However, the size of the inhibition zone alone does not fully reflect the antibacterial performance of sample, as other factors such as sustained release and contact-dependent activity may also contribute.

**Figure 4:**
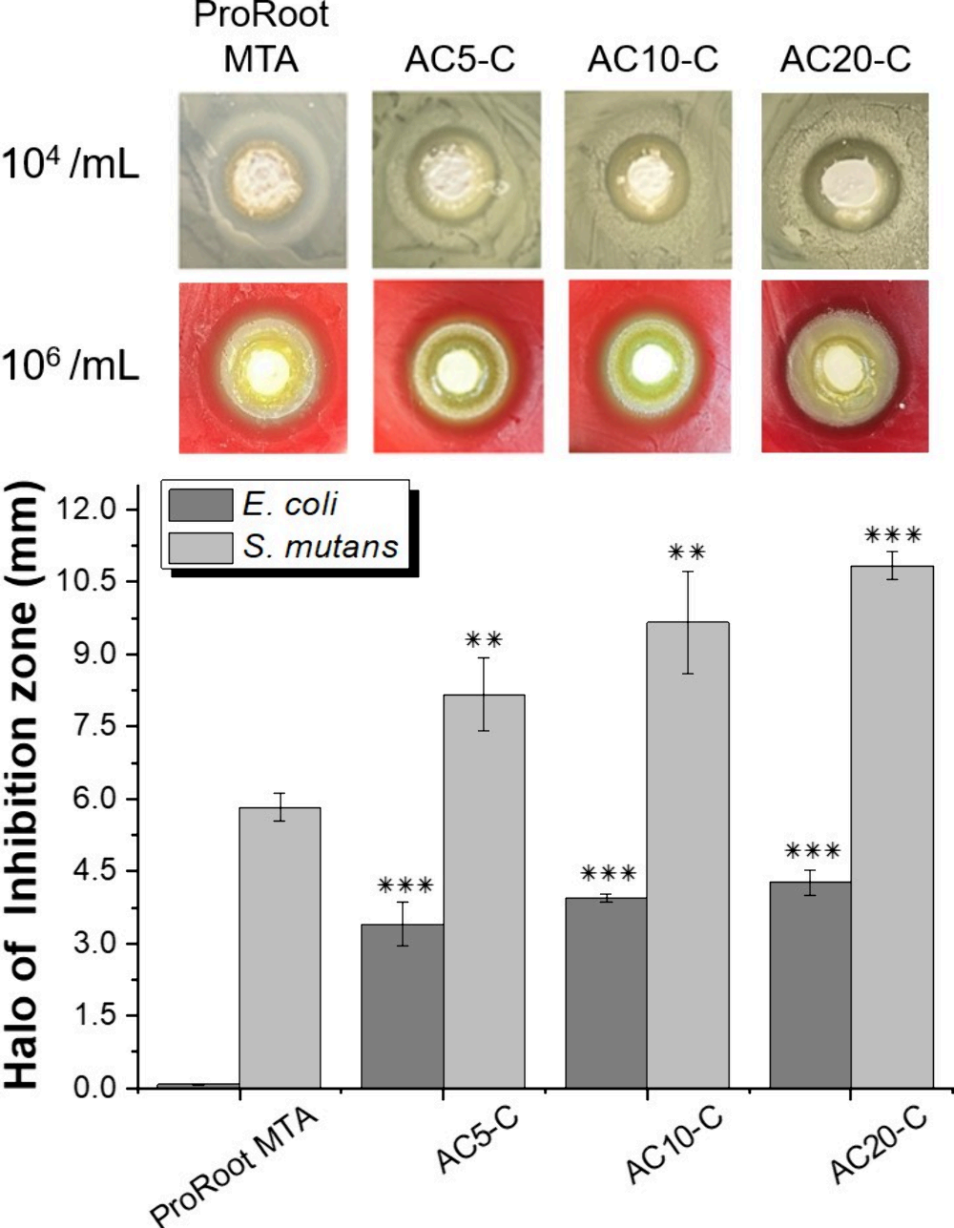
Assessment of inhibition zones by AC_*S*_-C: evaluating the efficacy of antimicrobial pulp-capping materials (AC_20_-C, AC_10_-C, and AC_5_-C) compared with that of ProRoot MTA against *E. coli* and *S. mutans*.

#### Assessment of Biofilm Inhibition and Disruption of AC_*S*_-C Dental-Pulp-Capping Materials

Preliminary experimental data confirmed that the AC_20_-C exhibited superior antimicrobial efficacy compared with ProRoot MTA and Biodentine. We examined their biofilm inhibition properties against the anaerobic Gram-negative bacterium *P. gingivalis* (Figure 5). None of the materials completely inhibited the bacteria at an optical density (OD_600_) of 0.4. At their respective MIC values, ProRoot MTA exhibited 24% inhibition, whereas AC_10_-C and AC_5_-C exhibited 54% and 39% inhibition, respectively. AC_20_-C demonstrated the highest inhibition, at 64%. Although the biofilm inhibition was not considerably strong, all three AC_*S*_-C materials had significantly lower *P* values (<0.01) than did ProRoot MTA.

**Figure 5:**
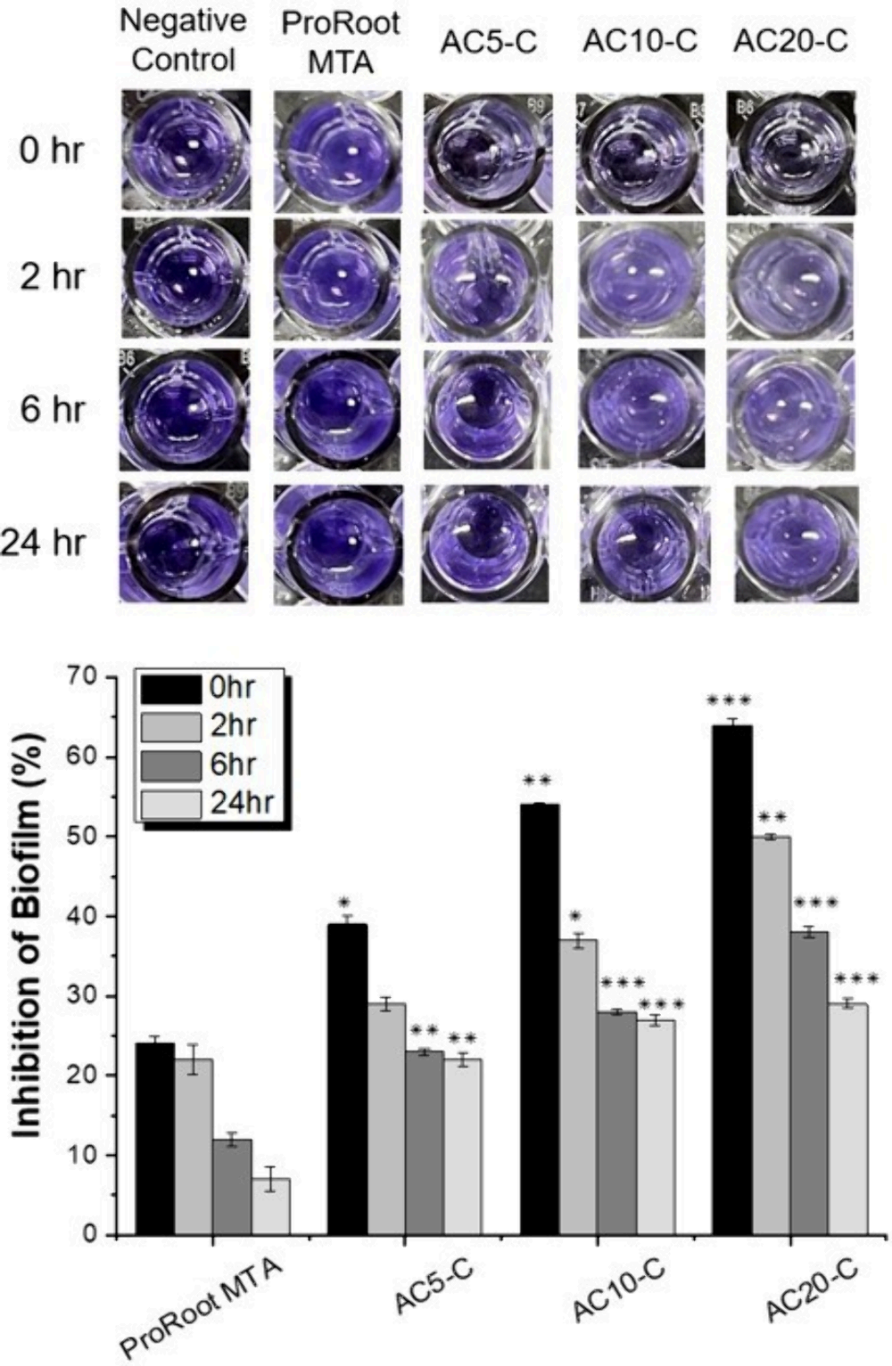
Evaluation of biofilm inhibition and disruption by AC_*S*_-C: comparing the efficacy of antimicrobial pulp-capping materials (AC_20_-C, AC_10_-C, and AC_5_-C) with that of ProRoot MTA against *P. gingivalis*.

The inhibition assays indicated that AC_20_-C exhibited the strongest biofilm inhibition among all tested materials. To further validate the efficacy of these materials against biofilms at various developmental stages, we investigated their effects on mature biofilms formed within 24 h. We examined early-, mid-, and late-stage biofilms, including preformed biofilms, to address the challenges of treating biofilms at different stages. For biofilms preformed for 2 h, AC_20_-C could disrupt 50% of the biofilm (*P* < 0.01), whereas ProRoot MTA reduced biofilm formation by approximately 22%. AC_10_-C and AC_5_-C achieved 37% and 29% disruption, respectively. As the biofilm formation time increased, AC_20_-C continued to demonstrate the highest disruption capability, achieving 38% disruption at 6 h and 29% at 24 h. However, none of the materials, including ProRoot MTA, AC_20_-C, AC_10_-C, and AC_5_-C, were effective against biofilms formed beyond 6 h. These findings indicated that although these pulp-capping materials exhibited varying degrees of biofilm inhibition against clinical strains of *P. gingivalis*, their effectiveness significantly decreased as the biofilms matured, particularly beyond 6 h of formation.

### Characterization of Physical Performance

#### Mechanical Properties and Setting Time of AC_*S*_-C Dental-Pulp-Capping Materials

Previous studies have indicated that the addition of chitosan can significantly prolong the setting time of calcium silicate-based cements.^51–53^ Chitosan interferes with the hydration reaction between calcium silicate and water, resulting in slower hardening and extending the setting time.

As shown in Figure 6A, incorporating a small amount of antimicrobial powder (AC_5_-C) had a minimal impact on setting time, which remain under 18 min. However, increasing the concentration of the antimicrobial powder extended the setting time to 20 min for AC_10_-C and 26 min for AC_20_-C. Despite this increase, all AC_*S*_-C samples still had a shorter setting time than ProRoot MTA, which required 36 min to set. Clinically, shorter setting time is preferred as it minimizes exposure to oral fluids and contamination during procedures.

**Figure 6:**
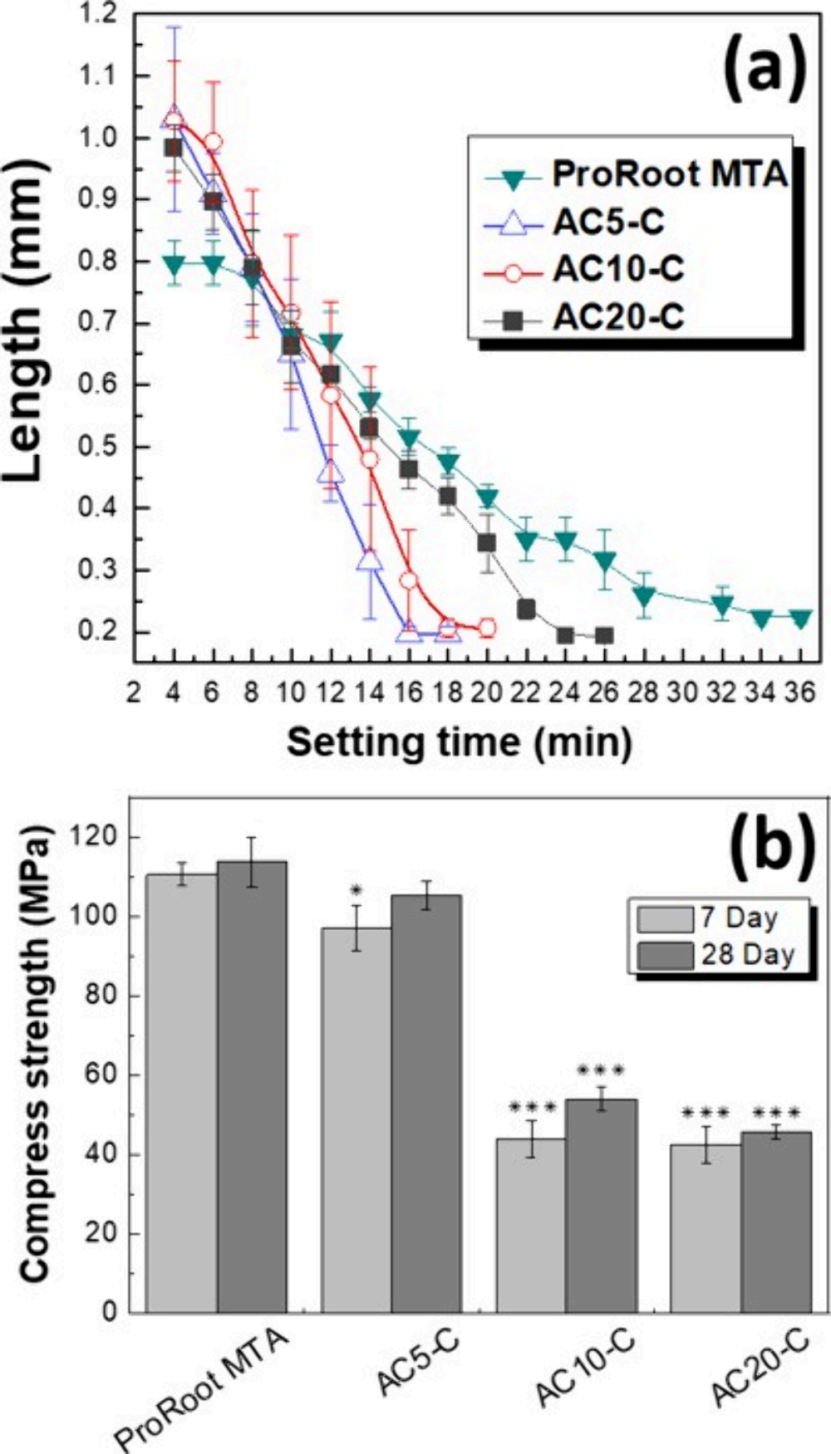
Physical properties and clinical feasibility of antimicrobial pulp-capping materials. (A) Comparative analysis of the setting time. (B) Compressive strength of antimicrobial bioceramic pulp-capping materials versus ProRoot MTA, *n* = 3.

Compressive strength (Figure 6B) was influenced by ion exchange, the formation of Ca­(OH)_2_, and the development of calcium silicate hydrate (C-S-H) during hydration. C-S-H is the principal product formed when tricalcium silicate (C_3_S) reacts with water, and it plays a crucial role in establishing early mechanical strength by binding particles together and filling pores within the material. After 7 days of hydration, ProRoot MTA exhibited an average compressive strength of 110.5 MPa, approximately 14 MPa higher than the 97.0 MPa observed for AC_5_-C. In contrast, AC_10_-C and AC_20_-C showed lower compressive strengths of 43.8 and 42.4 MPa, respectively. This difference is attributable to denser, more elastic microstructure of ProRoot MTA, which exhibits lower porosity and thus showed higher compressive forces. The decline in compressive strength in AC_10_-C and AC_20_-C may result from incomplete hydration and dilution effect caused by the higher content of antimicrobial powders, as well as a reduced water-to-powder ratio.^54^ High antimicrobial content can lead to dilution effects and incomplete hydration, resulting in a weaker microstructure.^55^ Thus, blends with high antibacterial content, such as AC_10_-C and AC_20_-C, may exhibit decreased compressive strength likely due to dilution effects and inhibited hydration, as excessive antimicrobial powders reduce the availability of C3S particles to fully react with water.

After 28 days of hydration, the compressive strength of ProRoot MTA increased slightly from 110.5 to 113.6 MPa. The compressive strengths of AC_5_-C, AC_10_-C, and AC_20_-C also increased to 105.3, 54.0, and 45.7 MPa, respectively. These increases reflect continued hydration and C-S-H development over time. However, high levels of chitosan-based antimicrobial additives likely interfered with the microstructural integrity, especially in AC_10_-C and AC_20_-C, despite extended curing. Although compressive strength is important, it may not be the most critical factor in these specific applications. While AC20-C is slightly below the ISO 9917–1 threshold of 50 MPa, AC10-C meets the requirement and AC5-C is comparable to ProRoot MTA, indicating sufficient mechanical performance for use as antimicrobial bioceramic pulp-capping agents.

#### FTIR Analysis of Functional Groups in AC_*S*_-C Dental-Pulp-Capping Materials

Figure 7 presents the FTIR spectral analysis of the synthesized materials to verify the interactions and retention of functional groups during the synthesis process. To assess the presence and chemical integrity of amino groups, the spectra were amplified in the relevant regions, confirming that the amino functionalities in AC_5_-C remained detectable and were not completely masked by the presence of C_3_S. This comparison helped determine whether the final product retained key absorption bands characteristic of chitosan in acetic acid. The FTIR spectra (Figure 7A) within the range of 4000–550 cm^–1^, illustrated chemical structure modifications in the antibacterial materials, confirming the successful fabrication of the pulp-capping materials. Absorption bands at 1600 cm^–1^ and in the 3950–3650 cm^–1^ range corresponded to −OH vibrations, indicative of water, alcohol, and Ca­(OH)_2_. Following the reaction of chitosan with acetic acid, the absorption band at 1150–1040 cm^–1^ weakened due to the deformational vibrations of −C–O–C– groups and the thermal degradation of CH, leading to molecular chain scission. In addition, the loss of OH stretching at 3450 cm^–1^ and changes in amino and amide groups within the 1560–1660 cm^–1^ range indicated molecular chain scission and degradation. These observations suggest that the synthesis process altered the chemical structure of the materials, particularly affecting the functional groups responsible for their antibacterial properties.

**Figure 7:**
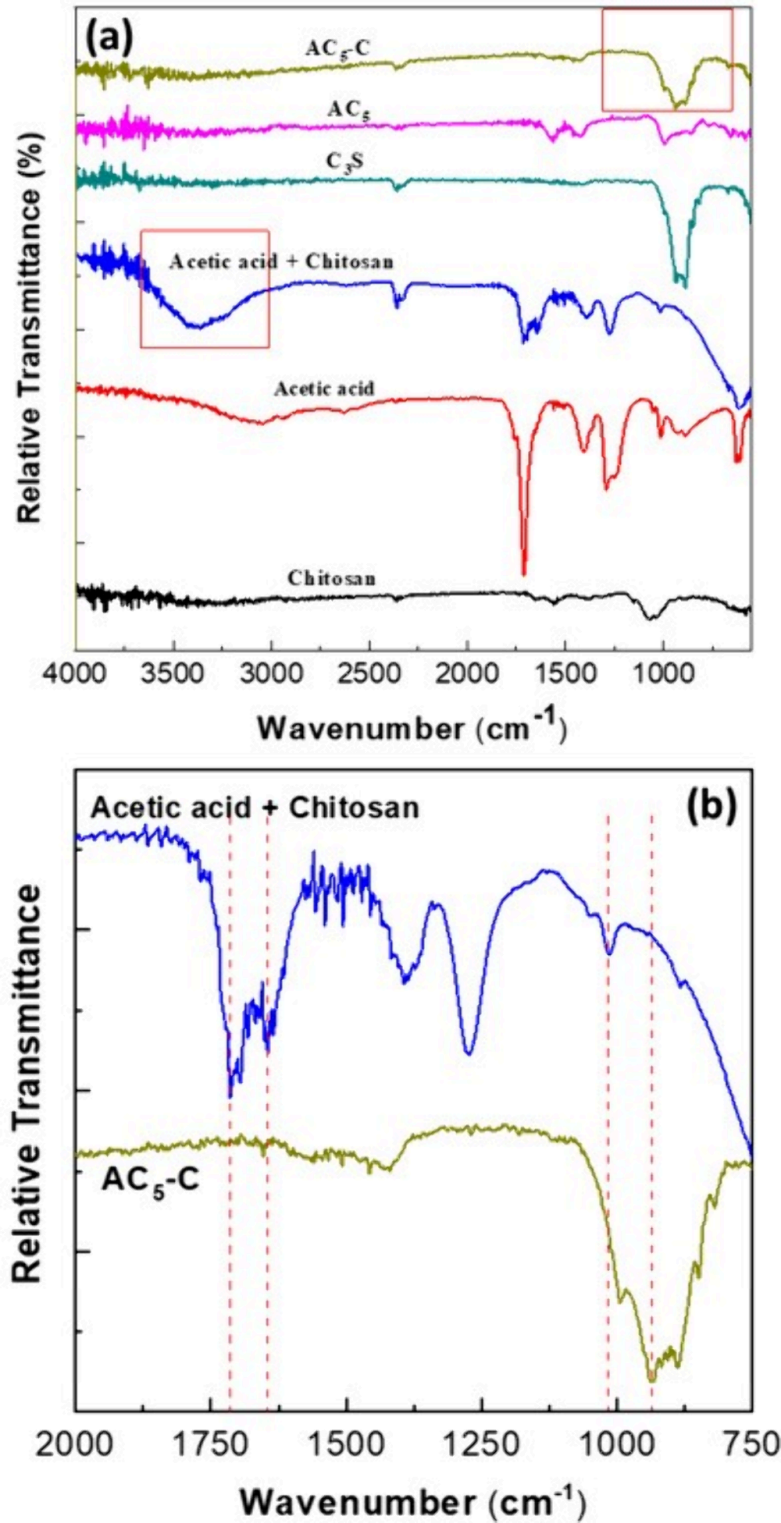
Comprehensive analysis of physical and chemical properties of AC_*S*_-C antimicrobial bioceramic pulp-capping materials. (A) FTIR spectra of all materials within the range of 750 to 1700 cm^–1^. (B) Magnified FTIR spectra analysis focused on amide group enhancement and structural rearrangement in antimicrobial pulp-capping material AC_5_-C and chitosan dissolved in acetic acid (Chitosan Solution).

When chitosan dissolved in acetic acid, marked changes were observed in its FTIR spectrum. Specifically, we magnified the spectrum (Figure 7B) and observed an increase in absorption at 1655 cm^–1^, corresponding to the C=O group, and at 1560 cm^–1^, corresponding to the −NH– group, indicating enhanced absorption for amide groups. Concurrently, the peak intensity for the amino group (NH_2_) at 1590 cm^–1^ decreased, suggesting reduced deacetylation. As the amide band absorbance at 1660 cm^–1^ increased, the amino band absorbance at 1590 cm^–1^ decreased. During the synthesis process, bond rearrangement may lead to macromolecular cross-linking and an increased gel fraction, with oxidation forming carbonyl groups, as evidenced by the increased absorbance at 1730 cm^–1^.^54^ For C_3_S, Si–O tensile vibrations were confirmed by the absorption band at 500–990 cm^–1^. Changes at approximately 1460 and 1485 cm^–1^ were associated with the presence of NO_3_^–^ and CO_3_^2–^ groups. Moreover, absorption changes occurred at approximately 961 cm^–1^ that were attributable to PO_4_^3–^ groups resulting from hydroxyapatite formation on C_3_S particles.^56^ The successfully synthesized antimicrobial powders contained amino and amide groups, as confirmed by absorption bands in the 1560–1660 cm^–1^ range and Si–O vibrations at approximately 990 cm^–1^. As illustrated in Figure 7, the antimicrobial pulp-capping materials, which combined antimicrobial powder with C_3_S, retained the −NH– (1600–1700 cm^–1^), Si–O (500–990 cm^–1^), and PO_4_^3–^ (961 cm^–1^) groups. This retention indicated successful functional group substitution and highlighted the ability of material to form Si–O, CaO, and surface PO_4_^3–^ groups under antibacterial conditions.

#### Evaluation of Crystal Structure in Dental-Pulp-Capping Materials

Phase analysis can be used to evaluate the efficacy of synthesis processes. In this study, a detailed compositional phase analysis was conducted on various materials, including pure C_3_S powders, AC_*S*_, and AC_*S*_-C. Figure 8 illustrates the XRD patterns used to observe phase changes before and after synthesis. Distinct peaks around 2θ = 20° corresponded to the amorphous structure of chitosan.^52^

**Figure 8:**
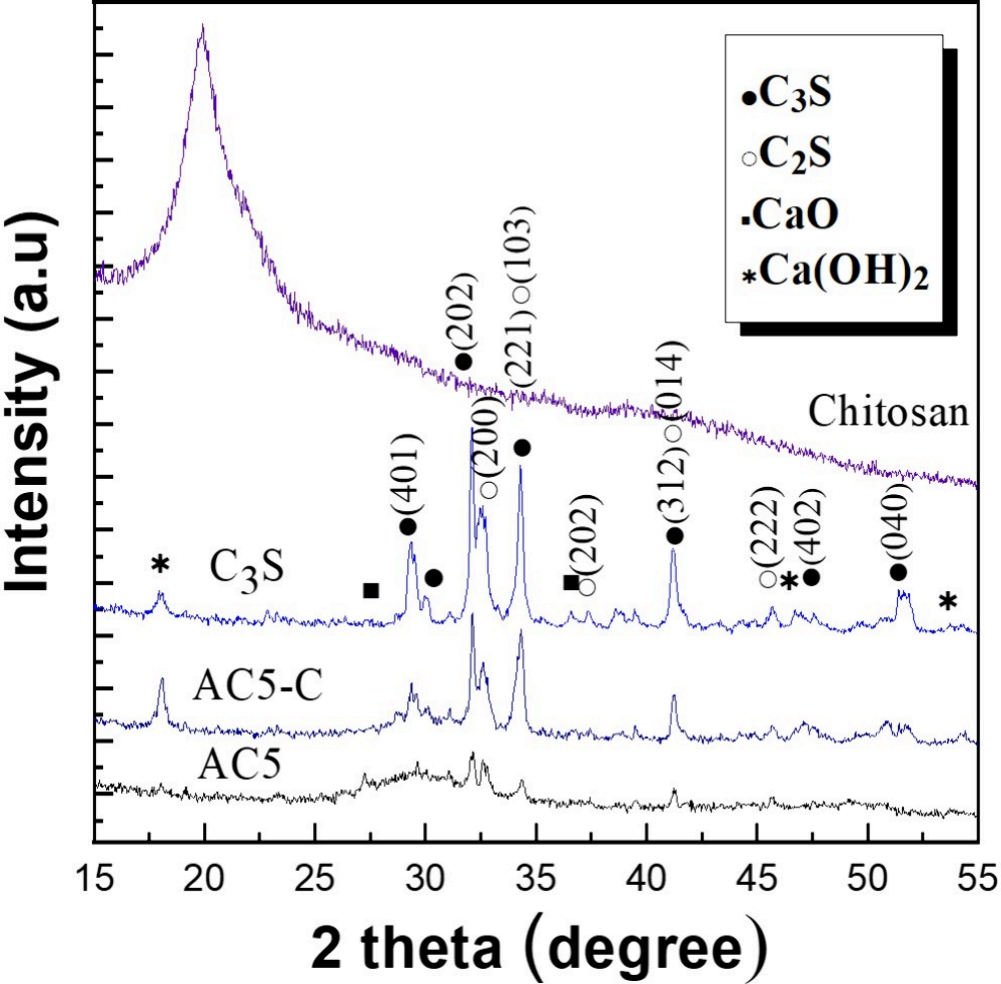
XRD patterns of different materials.

The synthesized material (AC_5_) retained some of the crystalline features of both C_3_S and C_2_S. However, new crystal structures also formed, causing subtle rightward shifts in the peaks associated with C_2_S and C_3_S. For instance, peaks for the C_2_S phase, initially observed at 32.7°, 34.6°, 37.7°, 41.5°, and 46.24°, were observed to shift rightward. Similarly, peaks for the C_3_S phase at 29.6°, 30.2°, 32.2°, 34.5°, 41.5°, 47.9°, 52°, and 56.6° also shifted due to the formation of new crystals. These shifts indicated structural changes occurring during synthesis, which in turn affected the properties of the material. A previous study^21^ suggested that higher calcium concentrations enhance the formation of reactive dentin and dense dentin bridges, thereby promoting stem cell proliferation and adhesion. The spectra analysis confirmed that AC_5_-C exhibited significant improvements in the C_3_S phase at 2θ = 32.2°, 34.5°, and 41° and in the C_2_S phase at 2θ = 32.7°, 34.6°, and 41.5°.

#### SEM-EDS Analysis

Figure 9, the top two rows present SEM images that illustrate the surface morphological changes of the antimicrobial pulp-capping material and ProRoot MTA after 7 and 28 days of immersion. Initially, the antibacterial material underwent a chemical transformation at 25 °C involving Ca^2+^ and hydroxyl ions from water, leading to the formation of Ca­(OH)_2_. After 7 days, this reaction resulted in a fibrous surface morphology, visible under SEM, with minor needle-like or flake-like structures indicative of hydroxyapatite formation. With prolonged immersion for 28 days, the fibrous structures became more pronounced, and the needle-like structures evolved into petal-shapes, indicating increased formation of C-S-H due to the ongoing reaction between C_3_S and H_2_O. By contrast, ProRoot MTA initially lacked significant fibrous surface morphology after 7 days of hydration. However, after 28 days, it developed fibrous structures and needle-like formations at the periphery, suggesting that prolonged hydration promotes the formation of both CSH and HA. These SEM observations align with the increased compressive strength observed after 28 days of hydration.

**Figure 9:**
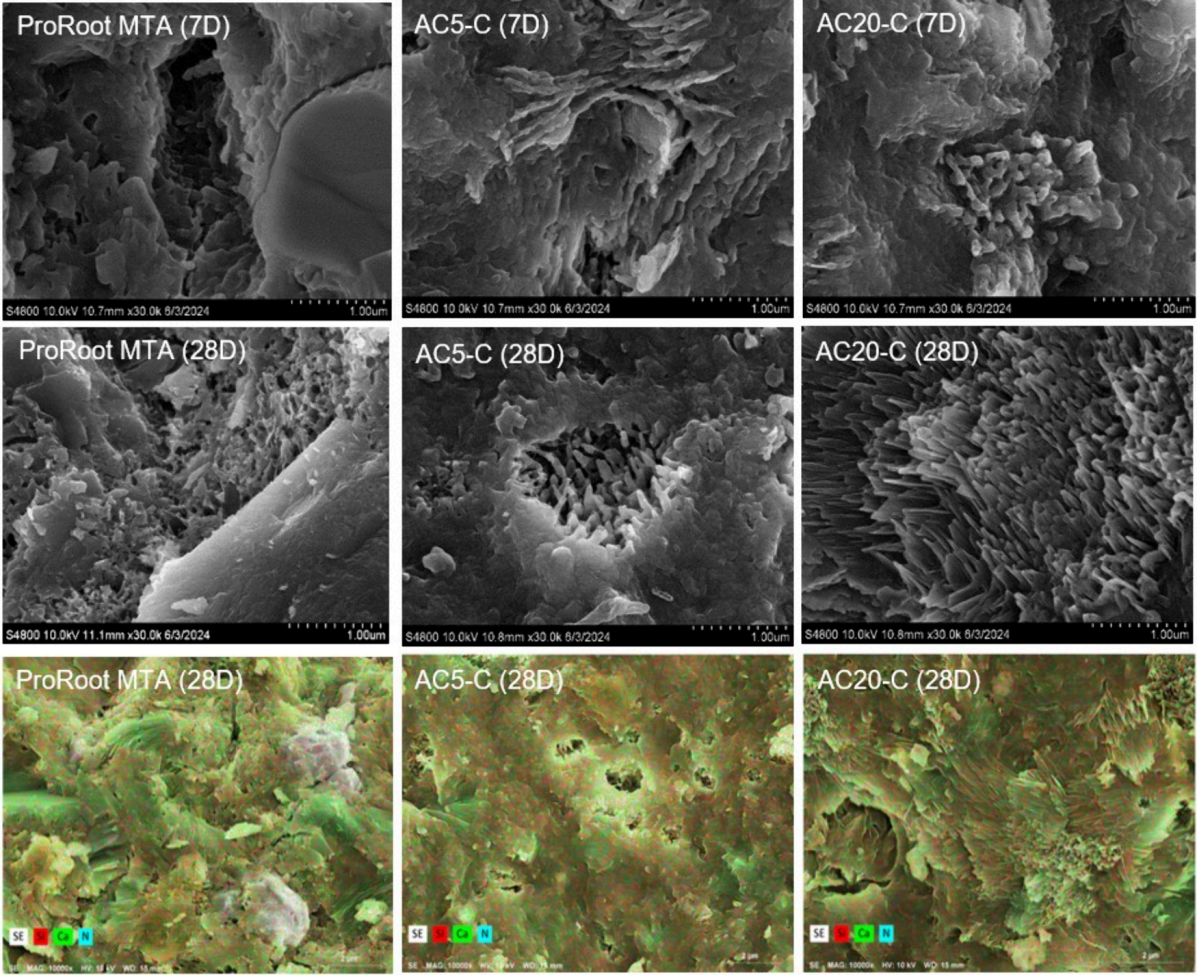
(top, middle) SEM images of ProRoot MTA, AC_20_-C, and AC_5_-C after 7 and 28 days of hydration. (bottom) EDS analysis, illustrating the chemical composition of ProRoot MTA, AC_20_-C, and AC_5_-C after 28 days of hydration.

EDX was conducted to better understand the chemical composition of the samples (Figure 9, bottom row). After 28 days of immersion in water, the calcium content in the antimicrobial pulp-capping materials AC_20_-C and AC_5_-C was measured at 72.26% and 70.58%, respectively, while the corresponding silicon contents were 27.74% and 29.42%, respectively. These results indicate an even distribution of calcium and silicon, suggesting that the high calcium ion content in these materials supports the formation of reactive dentin when the materials are in contact with dental pulp. Notably, ProRoot MTA, which contains Portland cement, CaO, and SiO_2_, had a calcium content of 73.06% and a silicon content of 26.94% after 28 days. This similarity in composition suggests that like ProRoot MTA, the synthesized antimicrobial pulp-capping materials facilitate the formation of dense dentin bridges.

## Discussion

Based on findings from previous studies,^38–40^ the present study revealed that during the hydration of C_3_S, OH^–^ ions are released, increasing the pH and creating an overly alkaline environment that hinders normal cell survival, metabolic activity, and proliferation. Although varying concentrations of chitosan were introduced, they did not fully mitigate the adverse effects of high pH on cell vitality, and excessively high concentrations of chitosan may further reduce cell viability. The metabolic activity of cells decreases as chitosan concentrations increase.^57^ Additionally, exposure of human liver cells to high concentrations of medium molecular weight chitosan nanoparticles (CSNP) (1% w/v nanoparticle concentration) has been shown to impaired cell membrane integrity.^58^ In contrast, lower concentrations of CSNP (10 and 100 μg/mL) were relatively nontoxic to mouse hematopoietic stem cells. Similarly, cell growth stabilized at a chitosan concentration of 0.1 mg/mL, while higher concentrations inhibited cell proliferation.^59^

Regarding the impact of an excessively alkaline environment on normal cell survival, it has been demonstrated that alkaline pH can enhance mineralization in human dental pulp cells and bone regeneration in human bone marrow mesenchymal stem cells, and that chitosan can promote osteoblast differentiation.^45,46,59^

In addition, the enhanced antibacterial effect can be attributed to stronger electrostatic interactions between the amino groups in chitosan and the anionic radicals present in bacterial cell walls. These interactions destabilize the cell wall, disrupt critical bacterial processes, and lead to cell death.^19,41^ Although AC_*S*_ compounds, which feature −OH and −NH_2_ groups that can alter cellular membranes and inhibit bacterial growth, exhibited strong antibacterial activity against both Gram-negative and Gram-positive bacteria, their effectiveness can vary depending on the structure of compounds and environmental conditions.^19,43^ The observed differences in antibacterial efficacy against *S. mutans* and *E. coli* are primarily due to differences in their cell membrane structures. *S. mutans* lacks a peptidoglycan layer, which affects its interaction with the protonated amino groups of chitosan, resulting in variations in antibacterial performance of the material against these bacteria.

Therefore, to effectively improve the antibacterial performance of the biomaterial, the experiment first synthesized high-concentration antimicrobial powders (AC_*S*_) and then physically mixed them with C_3_S. The resulting antimicrobial pulp-capping materials (AC_*S*_-C), containing 83.3% C_3_S, exhibited distinct new compound structures in the XRD and FTIR spectra shown in Figure 7 and 8. These findings suggest that beyond the initial chemical synthesis, the subsequent physical mixing process induces further molecular interactions and structural rearrangements, resulting in the formation of novel crystalline phases.

Among all formulations, AC_20_-C, which contained the highest concentration of antimicrobial powder, showed the strongest antibacterial performance. It achieved over 90% antibacterial efficacy against *E. coli* and *S. mutans*, with inhibition zones measuring 4.2 ± 0.25 mm and 10.8 ± 0.29 mm, respectively. Both AC_10_-C and AC_20_-C demonstrated antibacterial efficacy above 80%. However, in the inhibition of *P. gingivalis* biofilm formation, AC_20_-C showed significantly stronger suppression compared to AC_10_-C, particularly during the initial 0–6 h. These results indicate that AC_20_-C possesses superior antibacterial activity during the early stages of bacterial colonization. Given that pulp-capping materials are applied at the root apex, an area with a high likelihood of bacterial contact, the enhanced early stage antibacterial properties of AC_20_-C suggest its greater suitability for deep root canal therapeutic applications.

Notably, the reintroduction of C_3_S into the antimicrobial powders (AC_*S*_) to form AC_*S*_-C was associated with improved biocompatibility, as evidenced by the absence of significant cytotoxicity within 24 h, according to the MTT assay. This observation represents a potentially significant advancement in biomaterial design; however, such findings have been rarely reported in the existing literature. To clarify the mechanisms involved and confirm the reproducibility of this effect, further experimental studies are warranted. Collectively, these modifications not only preserve the alkaline microenvironment and promote 24 h cell proliferation but also result in a denser structure with an improved Ca/Si ratio, which contributes to the formation of a more robust and stable antibacterial layer.

Microstructural analysis using SEM and TEM revealed the in situ formation of hydroxyapatite (HAp) on the material surface following hydration. As the principal inorganic component of dentin and enamel, HAp enhances the bioactivity and tissue compatibility of the bioceramic. Its presence supports dental pulp stem cell adhesion and odontoblastic differentiation by providing a biomimetic microenvironment conductive to pulp tissue regeneration. Additionally, HAp deposition improves the sealing capability by filling surface micropores and cracks. The sustained release of calcium and hydroxide ions also contributes to mechanical reinforcement and creates a mildly alkaline environment that further supports antibacterial efficacy and cellular viability. In terms of mechanical requirements, root canal filling materials are subject to relatively modest demands. According to the ISO 9917-1 standard, the minimum compressive strength required for water-based dental cements is 50 MPa. Although the compressive strength of AC_20_-C is slightly below this threshold, it is primarily intended for use as a root-end filling material, where additional restorative materials are subsequently placed on top. In such cases, exceptionally high compressive strength is not essential, and other material properties, such as sealing ability and biocompatibility, may play a more decisive role in clinical performance. By comparison, AC_10_-C fulfills the ISO requirement, while AC_5_-C demonstrates a strength level comparable to that of ProRoot MTA, making it particularly suitable for use as a superficial capping material in pulp therapy. Taken together, these results indicate that compressive strength, while important, may not be the most critical factor in these specific applications, and overall, the tested materials exhibit adequate mechanical performance to support their intended clinical use. Additionally, the setting time was within 30 min, meeting standard clinical expectations. Moreover, the C_3_S powder developed in our laboratory (Ming-Kuan Biomedical Technology Co., Ltd., Hsinchu County, Taiwan) has been successfully scaled up for production and is currently being used in selected dental clinics. These initial applications highlight the translational potential of our AC_*S*_-C material for future endodontic therapies.

Futhermore, based on simulated test data for a dental pulp infection model and previous studies,^14,60^ we hypothesize that chitosan nanoparticles enhance the antibacterial effects of antimicrobial bioceramic pulp-capping materials. The present study demonstrated that although the addition of chitosan inhibited biofilm growth, the effectiveness declined with biofilm maturation. The ability to disrupt biofilms decreased from approximately 60% to 30% as the biofilm maturation time increased. This decline in effectiveness may be attributed to the increased resilience and vitality of bacteria in more mature biofilms, which render them more difficult to inhibit or disrupt. In addition, the inhibition mechanism may be limited, particularly under anaerobic conditions.

The contradictory results between suspension culture and agar diffusion methods often arise from differences in material-bacteria interactions and the diffusion behavior of antimicrobial agents. In suspension culture, the material is in direct and continuous contact with the bacterial suspension, enabling sustained release and uniform distribution of antimicrobial components, which enhances observable antibacterial effects over time.^61,62^ In contrast, the agar diffusion method depends on the ability of the active compounds to migrate through a semisolid medium. Materials that release ions slowly or have poor solubility, such as bioceramics or certain polymers, may exhibit limited diffusion, resulting in minimal or no inhibition zones despite strong bactericidal effects in liquid settings.^62,63^ Additionally, environmental factors like agar density and pH further influence this diffusion, making the method less sensitive for certain materials. Therefore, the apparent discrepancy is not due to inconsistent antimicrobial efficacy but rather to the limitations of the agar diffusion system in detecting slow-releasing or poorly diffusing substances.

Lastly, as the product is already in use and receiving feedback from dental practitioners, we include the following clinical observation based on practitioner reports: “According to feedback from clinical dentists, no postoperative reinfection or need for re-opening was observed after filling the root canal with AC_20_-C powder. In addition, radiographic images showed that the previously infected gingival tissue had returned to a healthy state approximately two months after AC_20_-C application, with no signs of inflammation.” Future IRB-approved clinical studies are currently in preparation to validate these observations and will be reported in subsequent publications.

## Conclusion

Antimicrobial bioceramic pulp-capping materials were developed by combining C_3_S powder with synthetic antimicrobial powders. A series of experiments was conducted to evaluate differences in antibacterial activity and biocompatibility by varying the concentration of chitosan solution and the mixing ratio between the antimicrobial powders and C_3_S. The effects of these variables on the physical, chemical, and mechanical properties of the materials were also examined. Our findings demonstrated that the AC_*S*_-C composite, formulated by blending antimicrobial powders (AC_*S*_) with 83.3% C_3_S, enhanced the surface characteristics and chemical composition of the chitosan-based bioceramics. This enhancement led to improved cell compatibility and facilitated the formation of a more stable and durable antibacterial interface, which supported cell proliferation within 24 h.

Among all tested formulations, AC_20_-C exhibited the most potent early stage antibacterial activity, achieving >90% inhibition against *E. coli* and *S. mutans*, and demonstrating strong suppression of *P. gingivalis* biofilm formation within 0–6 h. These properties suggest its suitability for root apex applications where rapid bacterial control is essential. In contrast, the AC_5_-C formulation, which incorporates a lower concentration of antimicrobial agents, achieved 70–80% antibacterial activity while retaining adequate compressive strength and a fast setting time. These characteristics indicate its potential for use in surface filling applications during root canal procedures, particularly where indirect contact with cells, bacteria, or blood is expected.

FTIR and XRD analyses confirmed the presence of amino, amide, Si–O, CaO, and PO_4_^3–^ groups, whereas EDX testing confirmed the presence of calcium and silicon components, with hydroxyapatite formation observed in the immersed samples. These results indirectly confirm that the newly synthesized antimicrobial bioceramic pulp-capping materials possesses good sealing properties and the potential to address issues related to pulp tissue infection and necrosis. Furthermore, the AC_*S*_-C composites exhibited in situ HAp formation, improved sealing ability, and sustained ion release, contributing to enhanced bioactivity, antibacterial performance, and cytocompatibility. The material also met ISO 9917-1 standard for compressive strength standards and demonstrated a clinically acceptable setting time.

## Data Availability

Data are available on request.

## References

[1] Moreira M. S., Sarra G., Carvalho G. L., Gonçalves F., Caballero-Flores H. V., Pedroni A. C. F., Lascala C. A., Catalani L. H., Marques M. M. (2021). Physical and biological properties
of a chitosan hydrogel
scaffold associated to photobiomodulation therapy for dental pulp
regeneration: An in vitro and in vivo study. BioMed. Res. Int..

[2] Wells, C. ; Dulong, C. ; McCormack, S. Vital Pulp Therapy for Endodontic Treatment of Mature Teeth: A Review of Clinical Effectiveness, Cost-Effectiveness, and Guidelines; Canadian Agency for Drugs and Technologies in Health: Ottawa, ON, 2019. https://europepmc.org/article/nbk/nbk546327?report=printable&client=bot&client=bot&client=bot. 31525010

[3] Ducret M., Montembault A., Josse J., Pasdeloup M., Celle A., Benchrih R., Mallein-Gerin F., Alliot-Licht B., David L., Farges J. C. (2019). Design
and characterization
of a chitosan-enriched fibrin hydrogel for human dental pulp regeneration. Dent. Mater..

[4] Wu M. K., Dummer P. M. H., Wesselink P. R. (2006). Consequences
of and strategies to
deal with residual post-treatment root canal infection. Int. Endod. J..

[5] Alhadainy H. A. (1994). Root perforations:
a review of literature. Oral Surg., Oral Med.,
Oral Pathol..

[6] Poggio C., Arciola C. R., Beltrami R., Monaco A., Dagna A., Lombardini M., Visai L. (2014). Cytocompatibility and
antibacterial
properties of capping materials. Sci. World
J..

[7] Salako N., Joseph B., Ritwik P., Salonen J., John P., Junaid T. A. (2003). Comparison of bioactive glass, mineral trioxide aggregate,
ferric sulfate, and formocresol as pulpotomy agents in rat molar. Dent. Traumatol..

[8] Tuna D., Ölmez A. (2008). Clinical long-term
evaluation of MTA as a direct pulp
capping material in primary teeth. Int. Endod.
J..

[9] Zhu N., Chatzistavrou X., Papagerakis P., Ge L., Qin M., Wang Y. (2019). Silver-doped
bioactive glass/chitosan hydrogel with potential application
in dental pulp repair. ACS Biomater. Sci. Eng..

[10] Torabinejad M., Hong C. U., Ford T. P., Kettering J. D. (1995). Antibacterial
effects of some root end filling materials. J. Endod..

[11] Kaur M., Singh H., Dhillon J. S., Batra M., Saini M. (2017). MTA versus
Biodentine: review of literature with a comparative analysis. J. Clin. Diag. Res..

[12] Bhavana V., Chaitanya K. P., Gandi P., Patil J., Dola B., Reddy R. B. (2015). Evaluation of antibacterial and antifungal
activity
of new calcium-based cement (Biodentine) compared to MTA and glass
ionomer cement. J. Conserv. Dent. Endod..

[13] Hiremath G. S., Kulkarni R. D., Naik B. D. (2015). Evaluation of minimal inhibitory
concentration of two new materials using tube dilution method: An:
in vitro: study. J. Conserv. Dent. Endod..

[14] Abusrewil S., Brown J. L., Delaney C., Butcher M. C., Tiba M., Scott J. A., Ramage G., McLean W. (2021). Chitosan enhances the
anti-biofilm activity of biodentine against an interkingdom biofilm
model. Antibiotics.

[15] de
Alvarenga E. S., de Oliveira C. P., Bellato C. R. (2010). An approach to understanding
the deacetylation degree of chitosan. Carbohydr.
Polym..

[16] Kou S. G., Peters L. M., Mucalo M. R. (2021). Chitosan: A review
of sources and
preparation methods. Int. J. Biol. Macromol..

[17] Beverlya R. L., Janes M. E., Prinyawiwatkula W., No H. K. (2008). Edible chitosan
films on ready-to-eat roast beef for the control of Listeria monocytogenes. Food Microbiol..

[18] Sarkar N. K., Caicedo R., Ritwik P., Moiseyeva R., Kawashima I. (2005). Physicochemical basis of the biologic
properties of
mineral trioxide aggregate. J. Endod..

[19] Ardean C., Davidescu C. M., Nemeş N. S., Negrea A., Ciopec M., Duteanu N., Negrea P., Duda-Seiman D., Musta V. (2021). Factors influencing the antibacterial activity of chitosan and chitosan
modified by functionalization. Int. J. Mol.
Sci..

[20] Bonson S., Jeansonne B. G., Lallier T. E. (2004). Root-end filling materials alter
fibroblasts differentiation. J. Dent. Res..

[21] Aeinehchi M., Eslami B., Ghanbariha M., Saffar A. S. (2003). Mineral trioxide
aggregate (MTA) and calcium hydroxide as pulp-capping agents in human
teeth: a preliminary report. Int. Endod. J..

[22] Luo Z., Li D., Kohli M. R., Yu Q., Kim S., He W. X. (2014). Effect
of Biodentine on the proliferation, migration and adhesion of human
dental pulp stem cells. J. Dent..

[23] Lan T. T. B., Tsai Y. C., Huang Z. Y., Chen Y. L., Hermosa G. C., Lu K. W., Chien C. C., Sun A. C. A. (2024). Modification
and characterization of tricalcium silicate bio-ceramic powders synthesized
by sol-gel process for potential application in dental treatment. Colloids Surf., A.

[24] How K. Y., Song K. P., Chan K. G. (2016). Porphyromonas
gingivalis: an overview
of periodontopathic pathogen below the gum line. Front. Microbiol..

[25] Leite M. L., Anselmi C., Soares I. P. M., Manso A. P., Hebling J., Carvalho R. M., de Souza
Costa C. A. (2022). Calcium silicate-coated porous chitosan
scaffold as a cell-free tissue engineering system for direct pulp
capping. Dent. Mater..

[26] Shaw R. G., Mitchell-Olds T. (1993). ANOVA for
unbalanced data: an overview. Ecology.

[27] Cooper, K. E. The theory of antibiotic inhibition zones. In Analytical Microbiology; Academic Press, 1963; pp 1–86. 10.1016/B978-1-4832-3129-7.50037-3.

[28] Mishra B., Wang G. (2017). Individual and combined effects of engineered peptides and antibiotics
on Pseudomonas aeruginosa biofilms. Pharmaceuticals.

[29] Kumar P., Nagarajan A., Uchil P. D. (2018). Analysis of cell viability by the
MTT assay. Cold Spring Harbor Protoc..

[30] Ghasemi M., Turnbull T., Sebastian S., Kempson I. (2021). The MTT assay: utility,
limitations, pitfalls, and interpretation in bulk and single-cell
analysis. Int. J. Mol. Sci..

[31] ISO 9917-1:2007: Dentistry—Water-Based Cements, Part 1: Powder/Liquid Acid-Base Cements, 2nd ed.; International Organization for Standardization: Geneva, 2007.

[32] Cardell C., Guerra I. (2016). An overview of emerging hyphenated SEM-EDX and Raman
spectroscopy systems: Applications in life, environmental and materials
sciences. TrAC Trends Anal. Chem..

[33] Niu F., Gu F., Zhao M., Gao Y., Tu W., Kou M., Pan W. (2023). Aggregation and growth
mechanism of ovalbumin and sodium carboxymethylcellulose
colloidal particles under thermal induction. Biomacromolecules.

[34] ISO 6876:2012: Dentistry—Root Canal Sealing Materials, 3rd ed.; International Organization for Standardization: Geneva, 2012.

[35] Kim D. H., Jang J. H., Lee B. N., Chang H. S., Hwang I. N., Oh W. M., Kim S. H., Min K. S., Koh J. T., Hwang Y. C. (2018). Anti-inflammatory
and mineralization effects of ProRoot
MTA and Endocem MTA in studies of human and rat dental pulps in vitro
and in vivo. J. Endod..

[36] ElReash A. A., Hamama H., Eldars W., Lingwei G., Zaen El-Din A. M., Xiaoli X. (2019). Antimicrobial activity
and pH measurement of calcium
silicate cements versus new bioactive resin composite restorative
material. BMC Oral Health.

[37] Queiroz M. B., Torres F. F. E., Rodrigues E. M., Viola K. S., Bosso-Martelo R., Chavez-Andrade G. M., Guerreiro-Tanomaru J.
M., Tanomaru-Filho M. (2021). Physicochemical,
biological, and antibacterial evaluation of tricalcium silicate-based
reparative cements with different radiopacifiers. Dent. Mater..

[38] Parirokh M., Torabinejad M. (2010). Mineral trioxide aggregate: a comprehensive
literature
review—part I: chemical, physical, and antibacterial properties. J. Endod..

[39] Khalil I., Naaman A., Camilleri J. (2016). Properties
of tricalcium silicate
sealers. J. Endod..

[40] Queiroz M. B., Torres F. F. E., Rodrigues E. M., Viola K. S., Bosso-Martelo R., Chavez-Andrade G. M., Guerreiro-Tanomaru J.
M., Tanomaru-Filho M. (2021). Physicochemical,
biological, and antibacterial evaluation of tricalcium silicate-based
reparative cements with different radiopacifiers. Dent. Mater..

[41] Helander I. M., Nurmiaho-Lassila E. L., Ahvenainen R., Rhoades J., Roller S. (2001). Chitosan disrupts
the barrier properties of the outer membrane of Gram-negative bacteria. Int. J. Food Microbiol..

[42] Hosseinnejad M., Jafari S. M. (2016). Evaluation of different
factors affecting antimicrobial
properties of chitosan. Int. J. Biol. Macromol..

[43] Chirkov S. N. (2002). The antiviral
activity of chitosan. Appl. Biochem. Microbiol..

[44] Keong L. C., Halim A. S. (2009). In vitro models
in biocompatibility assessment for
biomedical-grade chitosan derivatives in wound management. Int. J. Mol. Sci..

[45] Okabe T., Sakamoto M., Takeuchi H., Matsushima K. (2006). Effects of
pH on mineralization ability of human dental pulp cells. J. Endod..

[46] Kohn D. H., Sarmadi M., Helman J. I., Krebsbach P. H. (2002). Effects
of pH on human bone marrow stromal cells in vitro: implications for
tissue engineering of bone. J. Biomed. Mater.
Res..

[47] Goldberg M., Farges J. C., Lacerda-Pinheiro S., Six N., Jegat N., Decup F., Septier D., Carrouel F., Durand S., Chaussain-Miller C., DenBesten P., Veis A., Poliard A. (2008). Inflammatory
and immunological aspects of dental pulp repair. Pharmacol. Res..

[48] Erpaçal B., Adıgüzel Ö., Cangül S., Acartürk M. (2019). A general overview of chitosan and its use in dentistry. Int. Biol. Biomed. J..

[49] Sloan, A. J. Biology of the dentin-pulp complex. In Stem Cell Biology and Tissue Engineering in Dental Sciences; Academic Press, 2015; pp 371–378. 10.1016/B978-0-12-397157-9.00033-3.

[50] Goldberg M., Smith A. J. (2004). Cells and extracellular
matrices of dentin and pulp:
a biological basis for repair and tissue engineering. Crit. Rev. Oral Biol. Med..

[51] Panahi F., Rabiee S. M., Shidpour R. (2017). Synergic effect
of chitosan and dicalcium
phosphate on tricalcium silicate-based nanocomposite for root-end
dental application. Mater. Sci. Eng., C.

[52] Tonoli M. S., Beppu M. M. (2013). In situ X-ray diffraction
study of phase development
during hardening of β-tricalcium phosphate bone cements with
chitosan. Key Eng. Mater..

[53] Dessì M., Borzacchiello A., Mohamed T. H., Abdel-Fattah W. I., Ambrosio L. (2013). Novel biomimetic thermosensitive
β-tricalcium
phosphate/chitosan-based hydrogels for bone tissue engineering. J. Biomed. Mater. Res., Part A.

[54] Pawlak A., Mucha M. (2003). Thermogravimetric and
FTIR studies of chitosan blends. Thermochim.
Acta.

[55] Camiletti J., Soliman A. M., Nehdi M. L. (2013). Effect of nano-calcium
carbonate
on early-age properties of ultra-high-performance concrete. Mag. Concrete Res..

[56] Nikčević I., Jokanović V., Mitrić M., Nedić Z., Makovec D., Uskoković D. (2004). Mechanochemical
synthesis of nanostructured
fluorapatite/fluorhydroxyapatite and carbonated fluorapatite/fluorhydroxyapatite. J. Solid State Chem..

[57] Abusrewil S., Scott J. A., Alqahtani S. S., Butcher M. C., Tiba M., Kumar C., Mulvihill D. M., Ramage G., McLean W. (2024). The Effect
of Chitosan Incorporation on Physico-Mechanical and Biological Characteristics
of a Calcium Silicate Filling Material. Dent.
J..

[58] Loh J. W., Yeoh G., Saunders M., Lim L. Y. (2010). Uptake and cytotoxicity
of chitosan nanoparticles in human liver cells. Toxicol. Appl. Pharmacol..

[59] Singh M., Hiremath G., Bhat K. G., Naik B. (2020). Evaluation and Comparison
of the Effect of MTA, MTA Plus, Chitosan, and Their Conjugates on
Cell Viability of Human Periodontal Ligament Fibroblasts: An In Vitro
Study. J. Oper. Dent. Endod..

[60] del
Carpio-Perochena A., Kishen A., Felitti R., Bhagirath A. Y., Medapati M. R., Lai C., Cunha R. S. (2017). Antibacterial properties
of chitosan nanoparticles and propolis associated with calcium hydroxide
against single-and multispecies biofilms: an in vitro and in situ
study. J. Endod..

[61] Hossain T. J. (2024). Methods
for screening and evaluation of antimicrobial activity: A review of
protocols, advantages, and limitations. Eur.
J. Microbiol. Immunol..

[62] Balouiri M., Sadiki M., Ibnsouda S. K. (2016). Methods
for in vitro evaluating antimicrobial
activity: A review. J. Pharm. Anal..

[63] Steward C. D., Stocker S. A., Swenson J. M., O’Hara C. M., Edwards J. R., Gaynes R. P., McGowan J. E., Tenover F. C. (1999). Comparison of agar dilution, disk
diffusion, MicroScan,
and Vitek antimicrobial susceptibility testing methods to broth microdilution
for detection of fluoroquinolone-resistant isolates of the family
Enterobacteriaceae. J. Clin. Microbiol..

